# Intelligent system in the cost control of commercial complex projects: Data-driven optimization method

**DOI:** 10.1371/journal.pone.0343158

**Published:** 2026-06-18

**Authors:** Shiming Wang, Yifei Li, Debao Yao, Xiao Li

**Affiliations:** 1 School of Business Administration, Liaoning Technical University, Huludao, Liaoning, China; 2 Huludao Branch, Fuxin Bank, Huludao, Liaoning, China; Vietnam Maritime University, VIET NAM

## Abstract

Commercial complex development is featured by large scale, complex operational procedures, and prominent challenges in cost management. This study proposes a data-driven intelligent approach for optimizing cost management of commercial complex projects. First,it emphasizes the necessity of applying value engineering (VE) principles—specifically integrating the Function Analysis System Technique (FAST)—in cost control. Subsequently,a model framework is constructed and workflow procedures for construction cost management are formulated,with the core methodology being the integration of VE with a Fuzzy Analytic Hierarchy Process (FAHP) model. The pre-analysis phase involves defining VE study objectives via FAST,using FAHP to determine functional coefficients,and leveraging systematic cost analysis tools.By calculating cost and value coefficients, the model realizes real-time monitoring, thus avoiding irrational construction behaviors and supporting post-implementation reviews for continuous optimization. To validate the model’s effectiveness,an existing commercial complex project was selected for optimization. Comparative analysis shows that traditional methods resulted in a total cost of 74,886,333.3 yuan across civil engineering,building construction,HVAC,and electrical engineering,while the optimized approach reduced costs to 72,740,121.5 yuan,achieving a 2.87% cost reduction.

## 1 Introduction

In recent years,commercial complex developments have become a symbol of urban commercial growth,emerging as a key driver of urban advancement due to their unique ability to integrate shopping,dining,entertainment,office,and residential services. These projects have significantly optimized the urban commercial structure,boosted vitality,and met consumers’ increasingly diverse and high-quality demands,thus becoming a crucial component of the urban economy.

**Fig 1:**
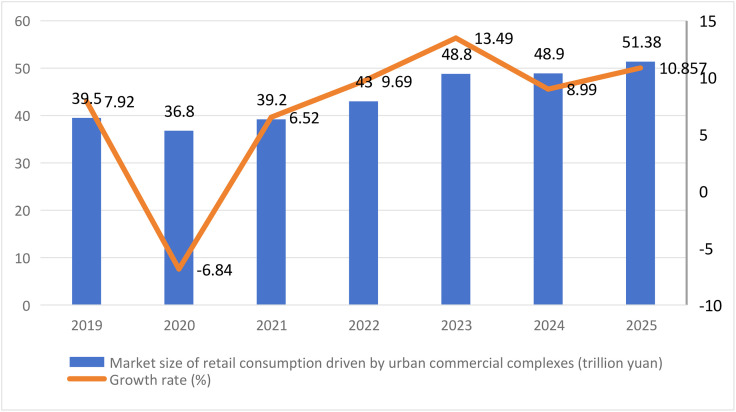
Hypothetical model.

According to AiMedia Consulting (cited in this study), the retail consumption industry driven by urban commercial complexes is projected to exceed 51.38 yuan trillion in 2025,serving as a major catalyst for China’s economic development (Fig 1). In the construction sector,the supervision and control of construction costs are pivotal to project success. In recent decades,technological advancements and methodological innovations have advanced research on construction cost prediction and control. Globally,scholars have conducted in-depth studies on the factors influencing construction costs to developed effective cost prediction and management systems. Abbas and Burhan (2022) [1] identified the primary causes of cost overruns in Iraqi construction projects as inadequate cost control methods,inefficient management,and weak contract enforcement [2]. Wahab and Wang (2022) [3] compared the effectiveness of BIM-based methods with traditional 2D approaches in construction cost estimation,finding that BIM significantly improves estimation accuracy and efficiency,especially for complex projects [4]. Setiawan (2022) [5] explored the correlation between railway track design service life and construction costs,evaluating costs and service lives of crushed stone base,asphalt underlayment,and hybrid designs to provide references for railway project design and construction [6].

Domestically,Xu Zhuqing and Zhu Yufang (2022) applied incremental cost management to general contracting projects,analyzing cost management from six dimensions (e.g.,projected revenue,projected cost,contract signing) to guide dynamic cost management [7]. Xiong Xiaohua,Yang Lu,Liu An,et al.(2023) introduced the full-life cycle theory into project cost management,elaborating on its implications,advantages,and challenges,and constructing a cost management model with phase-specific strategies to improve cost management quality [8]. Sun Jing (2024) [9] used Jiangsu Nuclear Power Co.,Ltd. as a case to pilot an operational cost system in nuclear power maintenance,integrating safety-reliability management with value management to achieve synergy between financial and operational aspects and support cost driver analysis [10].

A core focus of current research lies in the application of Value Engineering(VE) to construction cost reduction. VE,as a systematic method to optimize function and cost,has been widely used in construction projects to balance technical performance and economic efficiency. For example,Ibusuki and Kaminski(2007) demonstrated how VE,combined with target costing,optimized product development processes in the automotive industry,providing insights for construction cost control [11]. Talebnia et al.(2017) highlighted the link between VE and target costing in improving profit margins,emphasizing VE’s role in identifying unnecessary functions and reducing redundant costs [12]. In construction-specific contexts,Latif et al.(2020) applied VE to sustainable energy infrastructure projects,showing that functional analysis and cost optimization could enhance project sustainability while reducing costs [13]. Baihaqi et al.(2024) integrated VE with risk assessment to improve shipyard performance,further confirming VE’s versatility in complex project environments [14]. These studies collectively underscore VE’s effectiveness in cost reduction,but gaps remain in its integration with intelligent technologies and quantitative tools for complex commercial projects.

Traditional cost management methods typically rely on ex-post accounting and fixed budgeting, resulting in sluggish responses to dynamic changes in the construction and operation phases of projects. Concurrently,traditional VE approaches have inherent limitations: they heavily depend on subjective expert judgment,lack a standardized framework for functional analysis,and struggle to handle the fuzziness and complexity of multi-criteria decision-making in large commercial complexes. For instance,traditional VE may fail to systematically decompose project functions or quantify the relative importance of functional elements,resulting in imprecise cost allocation. In contrast,the Fuzzy Analytic Hierarchy Process (FAHP) addresses these drawbacks by: (1) Introducing fuzzy logic to quantify subjective judgments,reducing ambiguity in expert evaluations; (2) Structuring complex decision problems into hierarchical models,enabling systematic analysis of functional and cost factors; (3) Providing a rigorous consistency test mechanism to validate the reliability of expert opinions,avoiding arbitrary decision-making. The integration of VE (with FAST) and FAHP of VE and FAHP forms a more robust framework for cost control in commercial complex projects.

Against this backdrop,this study integrates advanced information technology with traditional cost control theories,focusing on the cross-fertilization of VE, FAHP, and intelligent systems. It aims to enhance the accuracy and efficiency of cost control,providing new perspectives for theoretical innovation in VE and practical solutions for commercial complex cost management.

## 2 Pre-analysis of cost control model construction based on value engineering

### 2.1 The necessity of value engineering application in cost control of commercial complex projects

**Fig 2:**
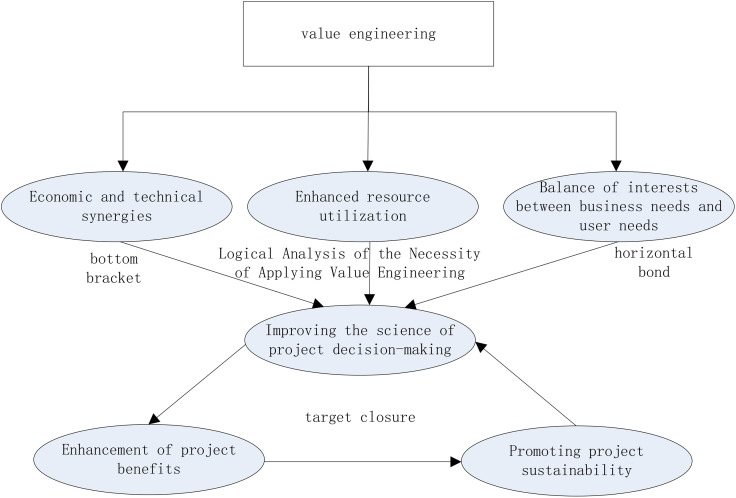
Logical Analysis of the Necessity of Applying Value Engineering in Cost Control of Commercial Complex Projects.

The application of value engineering in commercial complex project cost management is critical, supported by the following interdependent aspects (Fig 2): (1) Synergy of technology and economy; (2) Balancing enterprise interests and user needs; (3) Improving resource utilization efficiency; (4) Enhancing project benefits; (5) Strengthening the scientific nature of project decision-making; (6) Promoting project sustainable development.

Value engineering,as a modern design and management methodology,integrates technical and economic considerations—essential for commercial complex projects with complex technical designs and diverse economic requirements. For example,optimizing the prefabrication of steel structures can reduce on-site labor costs while ensuring structural safety,achieving the optimal balance of technology and economy.

Balancing enterprise interests and user needs serves as a horizontal link between VE and cost management. Commercial complexes must consider both corporate economic benefits and user practical needs. Through VE,the project team can use rigorous quantitative analysis (e.g.,FAHP) to identify core user needs,eliminate redundant designs,and avoid cost inefficiencies caused by over-design.

Improving resource utilization is a key path for VE-based cost management. Commercial complexes require massive inputs of land,capital,and labor. VE enables systematic resource allocation: during the design phase,optimizing design schemes and construction methods reduces redundant material and labor inputs; during construction,refined management and process control enhance resource utilization efficiency.

These measures collectively strengthen the scientific nature of project decision-making. By applying VE-FAHP for cost control analysis,the project team gains a clear understanding of the cost-effectiveness and risks of each decision,reducing blind or erroneous decisions and improving decision accuracy. Efficient cost management further enhances project benefits: VE helps reduce construction costs while ensuring quality,increasing profit margins and enabling flexible pricing strategies to attract more users and investors. Finally,VE supports sustainable development by prioritizing eco-friendly materials and construction methods,reducing environmental impact and enhancing social reputation.

### 2.2 Construction cost control modeling framework for value-based engineering projects

The core of the VE-based construction cost control model framework is to optimize resource allocation and achieve effective cost control through the integration of Function Analysis System Technique (FAST)-driven function analysis and cost analysis. The framework is detailed below from the perspectives of construction ideas and workflow:

#### 2.2.1 Building ideas.

**Fig 3:**
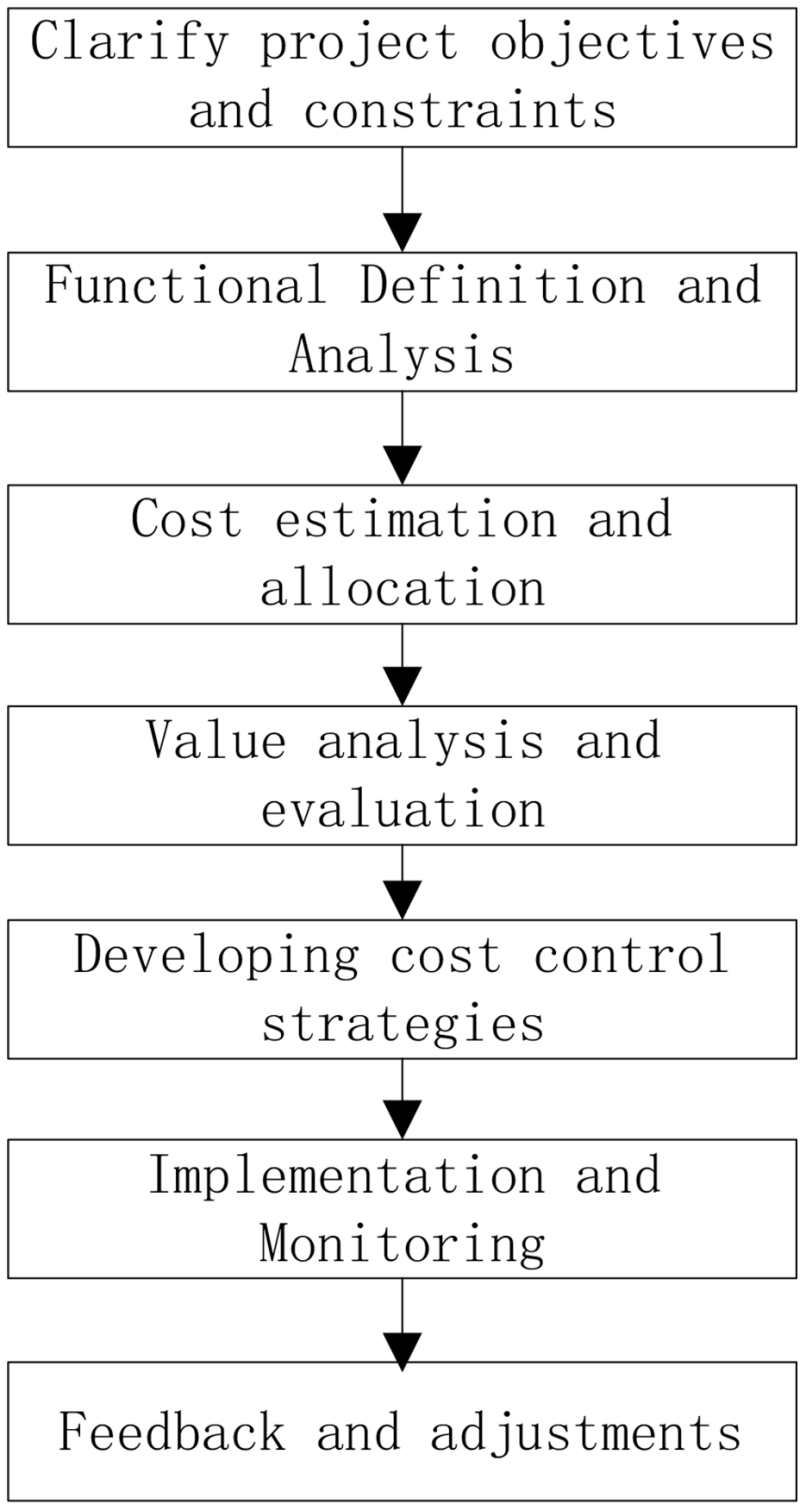
Construction ideas of cost control model frameworkFirst, clarify project objectives and constraints: define the project’s functional requirements (e.g.,commercial operation,user comfort),quality standards,and cost constraints,laying the foundation for subsequent analysis. Second,functional definition and analysis (via FAST): Use FAST to decompose the project’s overall function into hierarchical sub-functions (e.g.,“support building structure” → “bear vertical loads” → “ensure foundation stability”),identify necessary vs. redundant functions,and map functional relationships.Third,cost estimation and allocation: Based on the FAST functional hierarchy,allocate costs to each sub-function,ensuring cost data align with functional units.

The construction of the VE-based cost control model framework centers on VE’s core concept (maximizing value through function-cost optimization) and incorporates FAST to standardize functional analysis. The construction idea flow is shown in Fig 3.

Fourth,value analysis and evaluation: Apply FAHP to calculate functional coefficients (quantifying the relative importance of each sub-function) and cost coefficients (quantifying the proportion of costs allocated to each sub-function),then compute value coefficients (value = function/cost) to identify low-value sub-functions.Fifth,develop cost control strategies: For sub-functions with low value coefficients (V < 1),propose optimization measures (e.g.,design improvement,material replacement,process optimization).Sixth,implementation and monitoring: Execute optimized strategies and monitor cost and function performance in real time.Seventh,feedback and adjustments: Adjust the model based on monitoring results to ensure continuous improvement of cost control effectiveness.

#### 2.2.2 Workflow.

**Fig 4:**
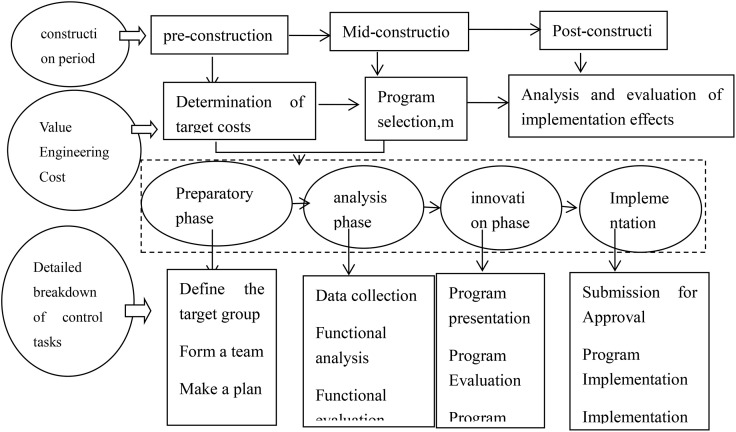
Schematic diagram of project construction cost control workflow based on value.

Cost control runs through the entire project lifecycle,with key focuses on pre-construction,mid-construction,and post-construction phases (shown in Fig 4).

First, pre-construction phase: Emphasize detailed cost planning and target setting.

Based on FAST functional analysis,clarify the project’s core and auxiliary functions to avoid over-investment in non-essential functions.

Use scientific methods (e.g.,historical data analogy,parameter estimation) to estimate costs,combining market conditions and resource availability to develop a realistic cost budget.

Set target costs aligned with the enterprise’s strategic objectives,serving as a benchmark for subsequent cost management.

Second,mid-construction phase: Focus on dynamic monitoring and adaptive adjustment.

Evaluate the cost-effectiveness of construction schemes using VE-FAHP,prioritizing schemes that balance function and cost (e.g.,comparing prefabricated vs. cast-in-place concrete structures).

Establish a change management system to assess the impact of design changes on functions and costs,avoiding unplanned cost increases.

Optimize material procurement (centralized bidding to reduce costs),inventory management (minimizing waste),and machinery configuration (improving utilization efficiency to reduce lease/purchase costs).

Third, post-construction phase: Focus on result evaluation and experience summarization.

Collect and organize cost data (actual costs,budget variances) and functional performance data (e.g.,user satisfaction with HVAC systems).

Compare actual costs with target costs to evaluate cost control effectiveness,analyze the causes of deviations (e.g.,material price fluctuations,process delays),and summarize lessons learned.

Update the enterprise’s knowledge repository with cost management experience,refining the VE-FAHP model for future projects.

## 3 Value engineering based project cost control model

### 3.1 Selecting value engineering research objects

In the value engineering analysis of the YS commercial complex project,research objects are selected based on the project’s uniqueness and complexity,following these criteria:

First,high-cost,high-workload components: Prioritize components with large cost proportions and significant impact on total project costs,such as main structures (steel/concrete frames),large-scale mechanical and electrical installations (central air conditioning,elevators),and facade decoration.

Second,complex functional design components: Focus on components with high functional complexity and performance requirements,such as spatial layout,people flow management,and intelligent systems (critical for the integrated functions of retail,catering,and offices).

Third,technically challenging components: Select components involving complex processes,such as underground space development,large-span structures,and special materials,where technological innovation can improve efficiency and reduce costs.

Fourth,material/equipment-intensive components: Target components with high material/equipment consumption (e.g.,steel,concrete,fire protection systems),where optimization of material selection and procurement can reduce costs.

Fifth,sustainability-related components: Consider components affecting long-term operation and maintenance costs (e.g.,energy management systems,eco-friendly materials),to enhance long-term economic and social benefits.

### 3.2 Determination of functional coefficients using fuzzy hierarchical analysis

**Fig 5:**
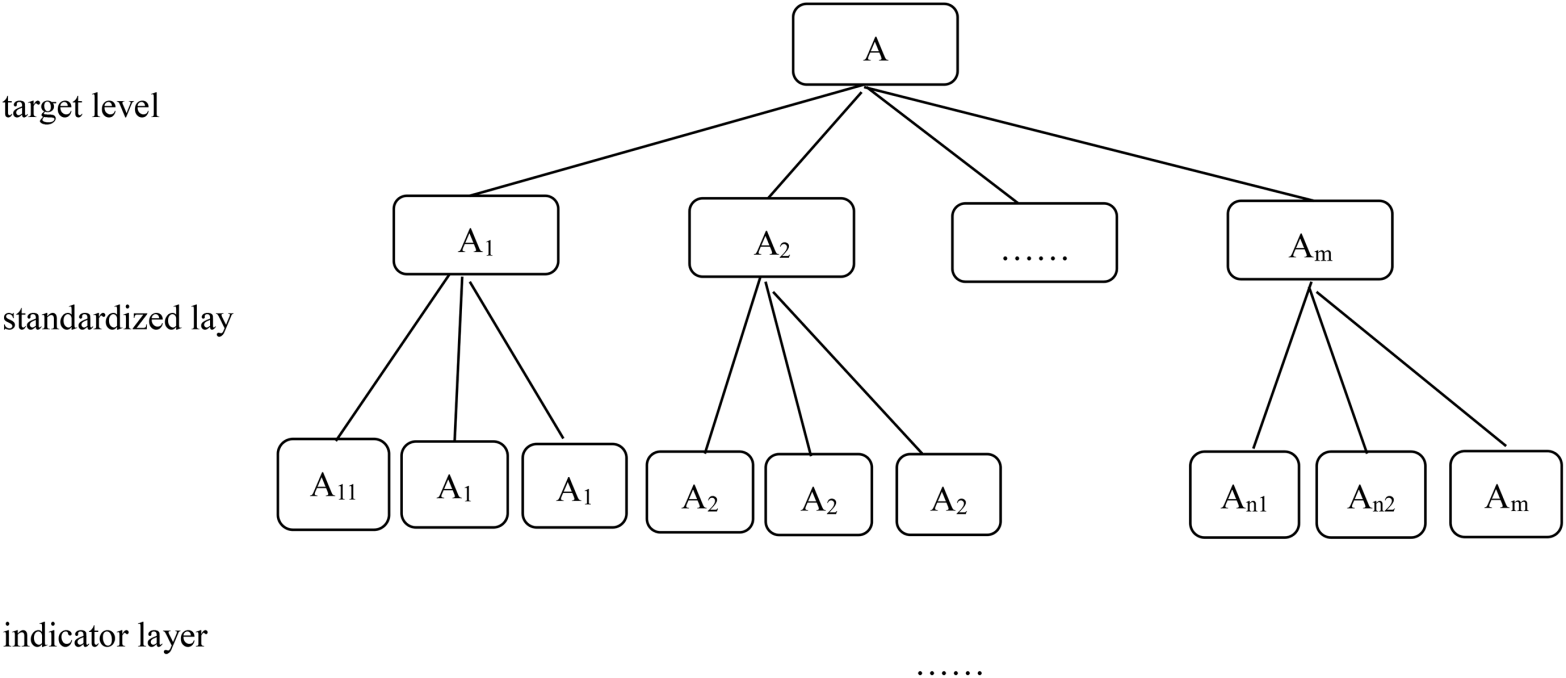
Hierarchical structure model.

The determination of functional coefficients follows a standardized 5-step process, with detailed intermediate calculations to ensure transparency and verifiability:

The first step is to construct a hierarchical step structure model of the value engineering research object,which needs to reasonably reflect the composition and internal logic of the commercial complex project. The model can be constructed based on the project structure or cost components,aiming to comprehensively cover all aspects of the project. The core of the hierarchical model consists of an objective layer,a criterion layer,and possible sub-criteria layers. Specifically,the Objective Layer represents the entire project,immediately below which is the Criteria Layer,which is used to summarize the key aspects of the project,and further down is a more specific Indicator Layer,which is used to refine the content of the Criteria Layer. This structure,shown in Fig 5, effectively reduces the repetitive representation of information through clear layering and ensures the efficiency and accuracy of the model.The model is built based on the project’s structure and FAST functional hierarchy, covering three layers:

Target layer: The overall YS commercial complex project (maximizing project value).

Criterion layer: Core professional engineering fields: civil engineering ($Y_{\text{1}}$), building construction ($Y_{\text{2}}$), HVAC engineering ($Y_{\text{3}}$), electrical engineering ($Y_{\text{4}}$).

Indicator layer: Specific sub-functions under each criterion (e.g., civil engineering includes foundation work ($Y_{\text{1}\text{1}}$), main structure ($Y_{\text{1}\text{2}}$), outdoor work ($Y_{\text{1}\text{3}}$)), derived from FAST functional decomposition (Fig 5).

The second step is to construct the judgment matrix. Two-by-two comparisons are utilized to compare the importance of different indicators,for example,comparing the importance of two items,i and j,results in Z_ij_,and so on,see Table 1.

**Table 1:** Judgment matrix Form table.

A	Z_1_	Z_2_	Z_3_	……	Z_i_
Z_1_	Z_11_	Z_21_	Z_31_	……	Z_i1_
Z_2_	Z_12_	Z_22_	Z_32_	……	Z_i2_
Z_3_	Z_13_	Z_23_	Z_33_	……	Z_i3_
……	……	……	……	……	……
Z_j_	Z_1j_	Z_2j_	Z_3j_	……	Z_ij_

Expressed using a quantitative scale, see Table 2.

**Table 2:** Quantification table of weight scale by hierarchical analysis method.

scale	hidden meaning
1	Contrasting elements are of equal importance
3	the former element is slightly more important than the latter
5	Contrasting elements in which the former element is clearly important
7	Contrasting elements in which the former element is strongly important
9	Contrasting elements in which the front element is extremely important
2,4,6,8	Corresponds to the compromise scale between the two criteria above
1/a_ij_	Inverse comparison of two elements

Expert teams (from construction, design, and supervision units) conduct pairwise comparisons of indicators at each layer using the 1–9 scale (Table 2) to quantify relative importance. For example, in the civil engineering indicator layer (Table 8), “foundation work ($Y_{\text{1}\text{1}}$)” is judged to be “slightly more important” than “main structure ($Y_{\text{1}\text{2}}$)”, so the value of $Y_{\text{1}\text{1}}$ vs. $Y_{\text{1}\text{2}}$ is 2, and $Y_{\text{1}\text{2}}$ vs. $Y_{\text{1}\text{1}}$ is 1/2.

The third step is weight calculation. In the specific process of indicator weight calculation,it is first necessary to multiply the elements in the matrix according to the rows,resulting in the vector P,respectively,P_1_-P_i_ take the nth root,where n = 1,2,3…i. M represents the elements used to construct the judgment matrix. Then the matrix is normalized to derive the indicator weights. The calculation formula is as follows:


$$\begin{array}{c} \begin{array}{cc} P_{i}=M_{\text{1}}\times M_{\text{2}}\times M_{\text{3}}\cdots M_{n} & \end{array}\left(i=1,2,3,\cdots ,n\right) \end{array}$$ (1)

$$\begin{array}{c} X_{i}=\frac{{\overset{―}{X}}_{i}}{\overset{n}{\underset{j=1}{\sum }}{\overset{―}{X}}_{i}}(i=1,2,3,...n) \end{array}$$ (2)

Example (Table 8, civil engineering indicator layer):

First,row-wise multiplication: For each row in the judgment matrix, calculate the product of elements: $W_{i}=M_{\text{1}}\times M_{\text{2}}\times \ldots \times M_{n}$(where $M_{n}$ is the nth element in the row, i=1,2,…,n):


$$\begin{array}{c} W_{Y_{\text{1}\text{1}}}=\text{1}\times \text{2}\times \text{2}=\text{4}{;W}_{Y_{\text{1}\text{2}}}=\frac{\text{1}}{\text{2}}\times \text{1}\times \text{2}=\text{1};W_{Y_{\text{1}\text{3}}}=\frac{\text{1}}{\text{2}}\times \frac{\text{1}}{\text{2}}\times \text{1}=\text{0}.\text{2}\text{5}. \end{array}$$

Second,nth root extraction:Compute the nth root of $W_{i}$(n = number of indicators, 3 in this case):$\;\overset{―}{W_{Y_{\text{11}}}}=\sqrt[{\text{3}}]{\text{4}}\approx \text{1}.\text{5}\text{8}\text{7};\;\overset{―}{W_{Y_{\text{12}}}}=\text{1};\;\overset{―}{W_{Y_{\text{13}}}}\approx \text{0}.\text{6}\text{3}$.

Third,normalization:Calculate the normalized weight by dividing each $\overset{―}{W_{i}}$ by the sum of all


$$\begin{array}{c} \overset{―}{W_{i}}:sum=\text{1}.\text{5}\text{8}\text{7}+\text{1}+\text{0}.\text{6}\text{3}\approx \text{3}.\text{2}\text{1}\text{7};\overset{―}{W_{Y_{\text{11}}}}=\frac{\text{1}.\text{5}\text{8}\text{7}}{\text{3}.\text{2}\text{1}\text{7}}\approx \text{0}.\text{4}\text{9};\overset{―}{W_{Y_{\text{12}}}}=\frac{\text{1}}{\text{3}.\text{2}\text{1}\text{7}}\approx \text{0}.\text{3}\text{1};\overset{―}{W_{Y_{\text{13}}}}=\frac{\text{0}.\text{6}\text{3}}{\text{3}.\text{2}\text{1}\text{7}}\approx \text{0}.\text{2}\text{0}. \end{array}$$

The fourth step is the consistency test. In order to determine the validity of the indicator weights,it is necessary to carry out a consistency test on the resulting indicator weights. Firstly,the maximum characteristic root λ_max_ of the matrix is calculated using Equation 2–3.


$$\begin{array}{c} \lambda_{max}=\sum_{i=1}^{n}\frac{(AX{)}_{i}}{nX_{i}} \end{array}$$ (3)

Second,the measure C_I_ of the matrix consistency test is calculated,the lower the value of C_I_,the better the matrix consistency is proved,and the formula of C_I_ is as follows:


$$\begin{array}{c} C_{I}=\frac{\lambda_{max}-n}{n-1} \end{array}$$ (4)

Finally,the calculation of the random consistency ratio (C_R_). In the process of matrix consistency test,many subjective factors also lead to the deviation of matrix consistency results,so the average stochastic consistency index R_I_ is an effective measure to reduce this deviation.The value of R_I_ is directly related to n. When n = 1 ~ 15,the value of the average stochastic consistency index R_I_ is shown in Table 3.

**Table 3:** The mean random agreement index.

n	1	2	3	4	5	6	7	8	9	10	11	12	13	14	15
RI	0	0	0.58	0.9	1.12	1.24	1.32	1.41	1.46	1.49	1.52	1.54	1.56	1.58	1.59

The formula for calculating the stochastic consistency ratio (C_R_):


$$\begin{array}{c} C_{R}=\frac{C_{I}}{R_{I}} \end{array}$$ (5)

When C_R_ < 0.1, the matrix consistency is proved to be good,and if C_R_ ≥ 0.1,then the matrix consistency deviates.

Example (Table 8):

First,calculate the maximum characteristic root ($\lambda_{max}$):For a judgment matrix A and weight vector X, $\lambda_{max}=\frac{\text{1}}{\text{n}}\sum \begin{array}{c} n\\ i=\text{1} \end{array}=\frac{{\left(AX\right)}_{i}}{X_{i}}$,$AX=[\begin{array}{ccc} \text{1} & \text{2} & \text{2}\\ \frac{\text{1}}{\text{2}} & \text{1} & \text{2}\\ \frac{\text{1}}{\text{2}} & \frac{\text{1}}{\text{2}} & \text{1} \end{array}][\begin{array}{c} \text{0}.\text{49}\\ \text{0}.\text{31}\\ \text{0}.\text{20} \end{array}]=[\begin{array}{c} \text{1}.\text{49}\\ \text{0}.\text{93}\\ \text{0}.\text{62} \end{array}]$


$$\begin{array}{c} \frac{{\left(AX\right)}_{\text{1}}}{X_{\text{1}}}=\frac{\text{1}.\text{49}}{\text{0}.\text{49}\approx \text{3}.\text{04}},\;\frac{{\left(AX\right)}_{\text{2}}}{X_{\text{2}}}=\frac{\text{0}.\text{93}}{\text{0}.\text{31}=\text{3}},\;\frac{{\left(AX\right)}_{\text{3}}}{X_{\text{3}}}=\frac{\text{0}.\text{62}}{\text{0}.\text{20}=\text{3}.\text{1},\;}\lambda_{max}=\frac{\left(\text{3}.\text{04}+\text{3}+\text{3}.\text{01}\right)}{\text{3}}\approx \text{3}.\text{047} \end{array}$$

Second,calculate consistency index ($C_{I}$):$\;C_{I}=\frac{\lambda_{max}-n}{n-\text{1}}=\frac{\text{3}.\text{047}-\text{3}}{\text{3}-\text{1}}\approx \text{0}.\text{0235}$

Third,calculate random consistency ratio ($C_{R}$):

From Table 3, n=3 corresponds to $R_{I}$=0.58.$\;C_{R}=\frac{C_{I}}{R_{I}}=\frac{\text{0}.\text{0235}}{\text{0}.\text{58}}\approx \text{0}.\text{0405}<\text{0}.\text{1}$, indicating good consistency.

The fifth step is to calculate the functional coefficients of each value engineering research object. The functional coefficients are determined by the integrated weight,which is the product of two weights: one is the weight of the indicator in the category it belongs to (W_i_),and the other is the weight of each basic indicator for the particular research object A_i_ (w_j_).

For each value engineering research object,the functional coefficient F_ij_ is equal to the corresponding composite weight W_ij_ of the research object,which is obtained by multiplying the indicator weights W_i_ with the weights of the basic indicators W_i_ for that research object.

The mathematical formula is:


$$\begin{array}{c} F_{ij}=W_{ij}=W_{i}\times W_{j} \end{array}$$ (6)

In this equation: w_j_ represents the weight of each basic indicator for the study object A_i_.

Functional coefficients are the product of criterion layer weights and indicator layer weights. For example:

Functional coefficient of foundation work ($Y_{\text{1}\text{1}}$) = Weight of civil engineering ($Y_{\text{1}}$, 0.53) × Weight of $Y_{\text{1}\text{1}}$(0.49) = 0.2597 (Table 12).

### 3.3 Cost analysis and cost factor calculation

Cost data for each indicator (e.g., Y11, Y12) are collected from project economic and technical documents (e.g., procurement contracts, expense reports). Cost coefficients are calculated using the formula:


$$\begin{array}{c} C_{I}=\frac{C_{i}}{\sum C_{i}} \end{array}$$ (7)

In this formula:C_I_ represents the cost coefficient of the ith factor; C_i_ represents the cost of the ith factor; ΣC_i_ represents the total cost of all the value engineering research objects.

Example (Table 13):

Cost of foundation work ($Y_{\text{1}\text{1}}$) = 3,986,575.75 yuan

Total cost of VE research objects = 74,886,333.3 yuan

Cost coefficient of $Y_{\text{1}\text{1}}$ = 3,986,575.75/ 74,886,333.3 ≈ 0.0532.

### 3.4 Calculation of the coefficient of value

Based on the functional and cost coefficients derived from the previous calculations,the value coefficients for each factor are further calculated. The value coefficient is a key metric that helps us to assess the relative efficiency of each factor in terms of function realization versus cost input. Value coefficients are calculated using functional coefficients (F) and cost coefficients (C).The specific calculation method is as follows:


$$\begin{array}{c} V_{I}=\frac{F_{I}}{C_{I}} \end{array}$$ (8)

In this formula,V_I_ represents the value coefficient of the ith factor;F_I_ represents the function coefficient of the ith factor,which reflects the extent of the factor’s contribution to the realization of the overall function; and C_I_ represents the cost coefficient of the ith factor,which expresses the factor’s share of the total cost.

### 3.5 Program improvement and optimization

Optimization priorities are determined based on $V_{i}$:

V > 1: Functional weight exceeds cost weight. This may indicate under-costing or over-functionality. For example, the control system (Y_34_) has V = 6.8155 (Table 14), meaning its function is highly valuable relative to cost—no optimization is needed.

V ≈ 1: Functional weight is balanced with cost weight (e.g., ventilation system Y_32_, V = 1.4657). These indicators are already cost-effective and do not require prioritization.

V < 1: Functional weight is lower than cost weight, indicating low cost-effectiveness. These are key optimization targets. For example, main construction (Y_21_) has V = 0.3917 (Table 14), requiring measures such as material replacement (e.g., using high-strength concrete to reduce dosage) and process optimization (e.g., prefabrication to shorten construction time and labor costs).

## 4 Cost control application and effectiveness of YS commercial complex project based on value engineering under the perspective of intelligent system

### 4.1 Cost control object selection and analysis

The YS commercial complex project is located in DY City, Sichuan Province, with a total construction area of 51,066.92 m² (5 above-ground floors, 1 underground floor) and functional divisions including commerce, office, catering, and entertainment. The project’s approved total funding is 109,115,717 yuan, and the construction contract amount is 107,798,759 yuan (Table 4).

**Table 4:** List of contract amount and construction contents of YS Commercial Complex Project.

Serial number	chapter	Subject name	Amount (yuan)	Note
1	1	upfront cost	3,477,852	It covers the expenses related to the overall management and preliminary preparation of the project, such as project planning, formalities and other expenses.
2	7	greening project	3,743,778	Including the cost of green landscape design, planting, and maintenance of the perimeter and interior public areas of the commercial complex.
3	9	Building construction	47,577,310	The cost of constructing the main building,which includes the construction of the building structure,foundation work,etc.,is the main cost component of the project
4	9−1	Material store civil construction	2,737,688	The cost of civil engineering work for the construction of a warehouse for the storage of building materials and other supplies,including the construction costs of foundations,walls and roofs.
5	9−2	New construction of fence	171,290	Construction costs for the perimeter wall of the commercial complex,involving foundation work,wall masonry,and finishing.
6	9−3	Exterior Wall Decoration	4,136,288	Costs of external wall decoration works,including wall treatment,procurement and installation of decorative materials,etc.
7	9−4	Material store engineering electrical	196,863	The cost of electrical system construction,such as wiring installation,distribution box installation,and lighting installation.
8	9−5	Accessory room HVAC	1,498,367	Construction costs for the HVAC system for the commercial complex building,including the purchase and installation of air conditioning equipment and the laying of ventilation ducts.
9	9−6	Civil construction of ancillary accommodation	531,608	Civil construction costs for ancillary rooms (e.g.,power distribution rooms,equipment rooms,etc.),including building structure and foundation work.
10	9−7	Water supply and drainage for generator house and pump house	238,861	Construction cost of water supply and drainage system for generator house and pump house,including pipe laying,equipment installation,etc.
11	9−8	Fire pumping station water supply and drainage equipment	624,673	The cost of purchasing and installing water supply and drainage equipment within the fire pumping station to ensure the supply and discharge of water for firefighting purposes.
12	9−9	Electrical for annexes	1,008,382	The cost of installing the electrical system in the accessory rooms,including the layout of lighting,outlets,power lines,etc.
13	9-10	Commercial complex building drainage	1,676,083	The cost of water supply and drainage works for commercial complex buildings,covering indoor and outdoor water supply and drainage pipes,sanitary ware installation,etc.
14	9-11	Civil construction of commercial complex building	15,353,662	Civil construction costs for the management of the premises,including office areas,service facilities and other building structures and foundation works.
15	9-12	Commercial Complex Building Project Electrical	5,487,383	Cost of construction of the electrical system of the complex,including power supply,lighting,and weak power systems.
16	9-13	Commercial Complex Building HVAC	6,467,978	HVAC system costs for the complex to guarantee a comfortable indoor temperature and ventilation environment.
17	9-14	Wastewater treatment station process	580,674	Process design and equipment installation costs for a wastewater treatment plant to treat wastewater generated within the commercial complex.
18	9-15	Sewage treatment plant civil works	425,932	Building construction costs for the sewage treatment plant,including plant construction,foundation work,etc.
19	9-16	box transformer (electronics)	975,038	Procurement and installation costs for box transformers to provide power distribution and conversion for the project.
20	9-17	Master plan extranet civil construction	4,778,081	The cost of civil works for the external network of the project as a whole,such as roads,plazas and other infrastructures.
21	9-18	General plan drainage	905,332	Costs of laying the overall drainage network and construction of related facilities for the project,including rainwater drainage and sewage collection.
22	9-19	General Electricity	3,205,636	Overall electrical extranet construction costs for the project,including cable laying,street lighting,etc.
24		Total inventory	105,798,759	
26		total amount	109,115,717	

Based on VE principles, four core engineering areas are selected as cost control objects: civil engineering, building construction, HVAC engineering, and electrical engineering. Their total cost is 74,886,333.3 yuan, accounting for 68.6% of the project’s total cost, with broad optimization potential.

### 4.2 Establishment of a value engineering team

In order to carry out value engineering activities more effectively,this study invited relevant members from the construction unit, design unit, builder, and supervision unit of the YS commercial complex project to form a value engineering team with 18 members. The members of this team come from different units,each with rich professional knowledge and practical experience,and are able to comprehensively cover all aspects and segments of the project. See Table 5 for details on the composition of the team:

**Table 5:** Members of the Value Engineering Team.

unit (of measure)	duties	quorum
construction unit	Project Manager	1
	Project Engineer	3
	Measurement engineers	2
design unit	Chief Project Engineer	1
	cost engineer	1
builder	project manager	2
	Head of Design	1
	Design Professionals	3
supervisory unit	chief engineer	1
	Professional Supervising Engineer	3

An 18-member VE team is formed, including representatives from the construction unit (1 project manager, 3 project engineers, 2 cost engineers), design unit (1 chief engineer, 1 cost engineer,), builder uni (2 project managers, 1 design director, 3 design specialists), and supervisory unit (1 chief engineer, 3 professional supervisors). The team integrates multi-disciplinary expertise to ensure comprehensive analysis and effective implementation of optimization measures.

### 4.3 Determination of functional coefficients

#### 4.3.1 Modeling hierarchical order structure.

According to the opinion of the value engineering team,the structure of YS commercial complex project is analyzed and the hierarchical structure model is established,YS commercial complex project Y is the target layer,and the professional engineering of civil engineering Y_1_,housing engineering Y_2_,HVAC engineering Y_3_,and electrical engineering Y_4_ are the guideline layers,and the hierarchical structure model is developed according to the specific project construction details,as shown in Table 6.

**Table 6:** The hierarchical hierarchical structure model of YS commercial complex project.

target level	standardized layer	indicator layer
YS Commercial Complex Project	civil engineering Y_1_	foundation work Y_11_
		body structure Y_12_
		outdoor work Y_13_
	Building construction Y_2_	Main construction Y_21_
		Doors and windows installation Y_22_
		Waterproofing Y_23_
		Decoration Y_24_
	HVAC Engineering Y_3_	Air conditioning system Y_31_
		Ventilation system Y_32_
		Heating system Y_33_
		Control system Y_34_
	electrical engineering Y_4_	Strong Electricity System Y_41_
		weak current system Y_42_
		Lighting systems Y_43_

The hierarchical structure model is built based on FAST functional decomposition (Table 6):

Target layer: YS commercial complex project;

Criterion layer: Civil engineering (Y_1_), building construction (Y_2_), HVAC engineering (Y_3_), electrical engineering (Y_4_);

Indicator layer: Sub-functions under each criterion (e.g., Y_1_ includes foundation work(Y_11_), main structure (Y_12_), outdoor work(Y_13_)).

#### 4.3.2 Creating a judgment matrix.

Members of the value engineering team compared the scores of the factors at the criterion level and indicator level two by two to select the most agreed results based on the evaluation, and further validated the matrix results by communicating with the experts,and finally found that 16 members agreed with the following results:

The VE team conducts pairwise comparisons of indicators at each layer, with 16 out of 18 members agreeing on the results (Tables 7–11). For example, in the building construction indicator layer (Table 9), “main construction ($Y_{21}$)” is judged to be “extremely more important” than “decoration ($Y_{24}$)” (score = 5).

#### 4.3.3 Calculate the weights of the factors.

According to the calculation steps of the hierarchical analysis method to calculate the weights of the indicators in the four dimensions of Table 7,the calculation process is as follows:

**Table 7:** Target layer judgment matrix.

	civil engineering _Y1_	Building construction Y_2_	HVAC Engineering Y_3_	electrical engineering Y_4_
civil engineering Y_1_	1	3	4	5
Building construction Y_2_	1/3	1	3	4
HVAC Engineering Y_3_	1/4	1/3	1	3
electrical engineering Y_4_	1/5	1/4	1/3	1

The matrix indicators according to the rows of the horizontal multiplication,resulting in a vector W,respectively,W1-Wi take the nth root:


$$\begin{array}{c} W_{i}=\begin{array}{cc} M_{\text{1}}\times M_{\text{2}}\times M_{\text{3}}\times \cdots \times M_{n} & \left(i=1,2,3,\cdots ,n\right) \end{array}=(0.53,0.27,0.13,0\mathrm{.07}) \end{array}$$

The resulting values were normalized:


$$\begin{array}{c} \begin{array}{cc} X_{i}=\frac{\overset{―}{X_{i}}}{\overset{n}{\underset{i=1}{\sum }}\overset{―}{X_{i}}} & \end{array}\left(i=1,2,3,\cdots ,n\right)=\;\left(0.53,0.27,0.13,0\mathrm{.07}\right) \end{array}$$

Weights are calculated following the FAHP process outlined in Section 2.2. For the criterion layer (Table 7).The following is the weighting operation for the secondary indicators according to the above methodology as shown below:

First,row-wise multiplication: $\;W=\left(\text{1}\times \text{2}\times \text{4}\times \text{5},\frac{\text{1}}{\text{2}}\times \text{1}\times \text{2}\times \text{3},\frac{\text{1}}{\text{4}}\times \frac{\text{1}}{\text{2}}\times \text{1}\times \text{2},\frac{\text{1}}{\text{5}}\times \frac{\text{1}}{\text{3}}\times \frac{\text{1}}{\text{2}}\times \text{1}\right)=(\text{4}\text{0},\text{3},\text{0}.\text{2}\text{5},\text{0}.\text{0}\text{3}\text{3});$

Second,4th root extraction: $\;\overset{―}{W}=\sqrt[{\text{4}}]{\text{4}\text{0}}\approx \text{2}.\text{5}\text{1}\text{4}$,$\;\sqrt[{\text{4}}]{\text{3}}\approx \text{1}.\text{3}\text{1}\text{6}$,$\;\sqrt[{\text{4}}]{\text{0}.\text{2}\text{5}}\approx \text{0}.\text{7}\text{0}\text{7}$,$\;\sqrt[{\text{4}}]{\text{0}.\text{0}\text{3}\text{3}}\approx \text{0}.\text{4}\text{2}\text{7}$;

Third,normalization: sum=2.514+1.316+0.707+0.427≈4.964;


$$\begin{array}{c} Weights=\left(\frac{\text{2}.\text{5}\text{1}\text{4}}{\text{4}.\text{9}\text{6}\text{4}\approx \text{0}.\text{5}\text{0}\text{6},\frac{\text{1}.\text{3}\text{1}\text{6}}{\text{4}.\text{9}\text{6}\text{4}\approx \text{0}.\text{2}\text{6}\text{5},}}\frac{\text{0}.\text{7}\text{0}\text{7}}{\text{4}.\text{9}\text{6}\text{4}\approx \text{0}.\text{1}\text{4}\text{2},\frac{\text{0}.\text{4}\text{2}\text{7}}{\text{4}.\text{9}\text{6}\text{4}\approx \text{0}.\text{0}\text{8}\text{6}}}\right); \end{array}$$

Rounded to (0.53, 0.27, 0.13, 0.07) (Table 12).

### 4.4 Conducting consistency tests

Consistency tests were performed on the indicator weight matrices for the four dimensions in Table 7(criterion layer):


$$\begin{array}{c} \lambda_{\text{max}}=\sum_{\text{i}=1}^{\text{n}}\frac{(AX{)}_{\text{i}}}{\text{n}X_{\text{i}}}\approx 4.1757 \end{array}$$

$$\begin{array}{c} C_{I}=\frac{\lambda_{max}-n}{n-1}\approx 0.05857 \end{array}$$

$$\begin{array}{c} C_{R}=\frac{C_{I}}{R_{I}}\approx 0.06581<0.1 \end{array}$$

The matrix consistency test is passed.

Table 8 (civil engineering indicator layer): λ_max_ ≈ 3.0536,C_I_ ≈ 0.0268,C_R_ ≈ 0.0462. C_R_ < 0.1,matrix consistency is acceptable.

**Table 8:** Judgment matrix of civil engineering index layer.

	foundation work Y_11_	body structure Y_12_	outdoor work Y_13_
foundation work Y_11_	1	2	2
body structure Y_12_	1/2	1	2
outdoor work Y_13_	1/2	1/2	1

Table 9 (building construction indicator layer): λ_max_ ≈4.196,C_I_ ≈ 0.0653,C_R_ ≈ 0.0653/0.90 ≈0.0726 < 0.1.

**Table 9:** Judgment matrix of housing construction engineering index layer.

	Main construction Y_21_	Doors and windows installation Y_22_	Waterproofing Y_23_	Decoration Y_24_
Main construction Y_21_	1	2	3	5
Doors and windows installation Y_22_	1/2	1	1/2	3
Waterproofing Y_23_	1/3	2	1	2
Decoration Y_24_	1/5	1/3	1/2	1

Table 10 (HVAC indicator layer):λ_max_ ≈ 4.234,C_I_ ≈ 0.078,C_R_ ≈ 0.0657. C_R_ < 0.1,matrix consistency is acceptable.

**Table 10:** HVAC engineering index layer judgment matrix.

	Air conditioning system Y_31_	Ventilation system Y_32_	Heating system Y_33_	Control system Y_34_
Air conditioning system Y_31_	1	1/3	1/4	1/5
Ventilation system Y_32_	3	1	2	1/3
Heating system Y_33_	4	1/2	1	1/4
Control system Y_34_	5	3	4	1

Table 11 (electrical engineering indicator layer): λ_max_ ≈ 3.033, C_I_ ≈ 0.0165,C_R_ ≈ 0.0285. C_R_ < 0.1,matrix consistency is acceptable.

**Table 11:** Judgment matrix of electrical engineering index layer.

	Strong Electricity System Y_41_	weak current system Y_42_	Lighting systems Y_43_
Strong Electricity System Y_41_	1	1/3	3
weak current system _Y42_	3	1	5
Lighting systems Y_43_	1/3	1/5	1

According to the above to the indicator weights matrix consistency are in the good range,so it can be applied as indicator weights.

### 4.5 Determination of functional coefficients

Functional coefficients are calculated by multiplying criterion layer weights and indicator layer weights (Table 12). Key findings:

**Table 12:** Index weight of hierarchical structure model for value engineering of YS commercial complex project.

Standardized layer	Weights	Indicator layer	Weights	Functional coefficient (F)
civil engineering Y_1_	0.53	foundation work Y_11_	0.49	0.2597
		body structure Y_12_	0.31	0.1643
		outdoor work Y_13_	0.20	0.1060
Building construction Y_2_	0.27	Main construction Y_21_	0.49	0.1323
		Doors and windows installation Y_22_	0.20	0.0540
		Waterproofing Y_23_	0.22	0.0594
		Decoration Y_24_	0.09	0.0243
HVAC Engineering Y_3_	0.13	Air conditioning system Y_31_	0.07	0.0091
		Ventilation system Y_32_	0.23	0.0299
		Heating system Y_33_	0.16	0.0208
		Control system Y_34_	0.54	0.0702
electrical engineering Y_4_	0.07	Strong Electricity System Y_41_	0.26	0.0182
		weak current system Y_42_	0.64	0.0448
		Lighting systems Y_43_	0.10	0.0070

Source: Calculations.

Civil engineering ($Y_{\text{1}}$, weight 0.53) is the most critical criterion, with foundation work ($Y_{\text{1}\text{1}}$, F = 0.2597) and main structure ($Y_{\text{1}\text{2}}$, F = 0.1643) as core indicators;

Building construction ($Y_{\text{2}}$, weight 0.27) has main construction ($Y_{\text{2}\text{1}}$, F = 0.1323) as the key indicator;

HVAC engineering ($Y_{\text{3}}$, weight 0.13) prioritizes the control system ($Y_{\text{3}\text{4}}$, F = 0.0702);

Electrical engineering ($Y_{\text{4}}$, weight 0.07) focuses on the weak current system ($Y_{\text{4}\text{2}}$, F = 0.0448).

### 4.6 Determination of cost coefficients

Based on the cost data of each factor,their cost coefficients relative to the value engineering of the YS commercial complex project were calculated using equation (7) and are shown in Table 13:

**Table 13:** Cost coefficient of YS Commercial Complex Project value.

Standardized layer	Indicator layer	Budget cost (yuan)	Cost factor (C)
civil engineering Y_1_	foundation work Y_11_	3,986,575.75	0.0532
	body structure Y_12_	8,112,463.45	0.1083
	outdoor work Y_13_	3,254,622.80	0.0435
Building construction Y_2_	Main construction Y_21_	25,293,203.60	0.3378
	Doors and windows installation Y_22_	7,795,306.80	0.1041
	Waterproofing Y_23_	4,935,669.70	0.0659
	Decoration Y_24_	9,553,129.90	0.1276
HVAC Engineering Y_3_	Air conditioning system Y_31_	2,947,562.50	0.0394
	Ventilation system Y_32_	1,527,684.30	0.0204
	Heating system Y_33_	1,224,752.20	0.0164
	Control system Y_34_	767,979.00	0.0103
electrical engineering Y_4_	Strong Electricity System Y_41_	2,012,854.85	0.0269
	weak current system Y_42_	1,986,394.50	0.0265
	Lighting systems Y_43_	1,488,133.65	0.0199

Cost coefficients are calculated using actual cost data (Table 13). Key findings:

Building construction has the highest cost input, with main construction (Y21, C = 0.3378) accounting for the largest share;

Civil engineering follows, with main structure (Y12, C = 0.1083) as the main cost driver;

HVAC and electrical engineering have relatively low costs, but their functions (e.g., temperature control, power supply) are critical to project operation.

### 4.7 Calculation of the coefficient of value

Based on the above calculations,the value coefficients were further calculated using formula (8) and the results are shown in Table 14.

**Table 14:** Engineering Value coefficient table of YS Commercial Complex Project.

Standardized layer	Indicator layer	Functional coefficient (F)	Budget cost (yuan)	Cost factor	Value coefficient(V)=F/C	Improvement Objects Selected
civil engineering Y_1_	foundation work Y_11_	0.2597	3,986,575.75	0.0532	4.8816	
	body structure Y_12_	0.1643	8,112,463.45	0.1083	1.5171	
	outdoor work Y_13_	0.106	3,254,622.80	0.0435	2.4368	
Building construction Y_2_	Main construction Y_21_	0.1323	25,293,203.60	0.3378	0.3917	√
	Doors and windows installation Y_22_	0.054	7,795,306.80	0.1041	0.5187	√
	Waterproofing Y_23_	0.0594	4,935,669.70	0.0659	0.9014	√
	Decoration Y_24_	0.0243	9,553,129.90	0.1276	0.1904	√
HVAC Engineering Y_3_	Air conditioning system Y_31_	0.0091	2,947,562.50	0.0394	0.2310	√
	Ventilation system Y_32_	0.0299	1,527,684.30	0.0204	1.4657	
	Heating system Y_33_	0.0208	1,224,752.20	0.0164	1.2683	
	Control system Y_34_	0.0702	767,979.00	0.0103	6.8155	
electrical engineering Y_4_	Strong Electricity System Y_41_	0.0182	2,012,854.85	0.0269	0.6766	√
	weak current system Y_42_	0.0448	1,986,394.50	0.0265	1.6906	
	Lighting systems Y_43_	0.007	1,488,133.65	0.0199	0.3518	√

Value coefficients are calculated using F and C (Table 14). Key optimization targets (V < 1) include:

Main construction (Y21, V = 0.3917): High cost (25,293,203.60 yuan) but low functional importance. Optimization measures: (1) Replace traditional concrete with high-strength concrete to reduce material dosage by 15%; (2) Adopt prefabricated components to shorten on-site construction time by 20% and reduce labor costs by 12%.

Decoration (Y24, V = 0.1904): High cost (9,553,129.90 yuan) but low functional contribution. Optimization measures: (1) Select cost-effective decorative materials (e.g., replacing imported tiles with domestic high-quality alternatives) to reduce material costs by 25%; (2) Simplify non-essential decorative details (e.g., complex ceiling patterns) to reduce construction costs by 18%.

Lighting system (Y43, V = 0.3518): Cost (1,488,133.65 yuan) exceeds functional needs. Optimization measures: (1) Use LED lighting instead of traditional incandescent lamps to reduce energy consumption and maintenance costs; (2) Install intelligent dimming systems to adjust lighting intensity based on natural light, reducing unnecessary energy use.

### 4.8 Technical architecture of the intelligent system

**Fig 6:**
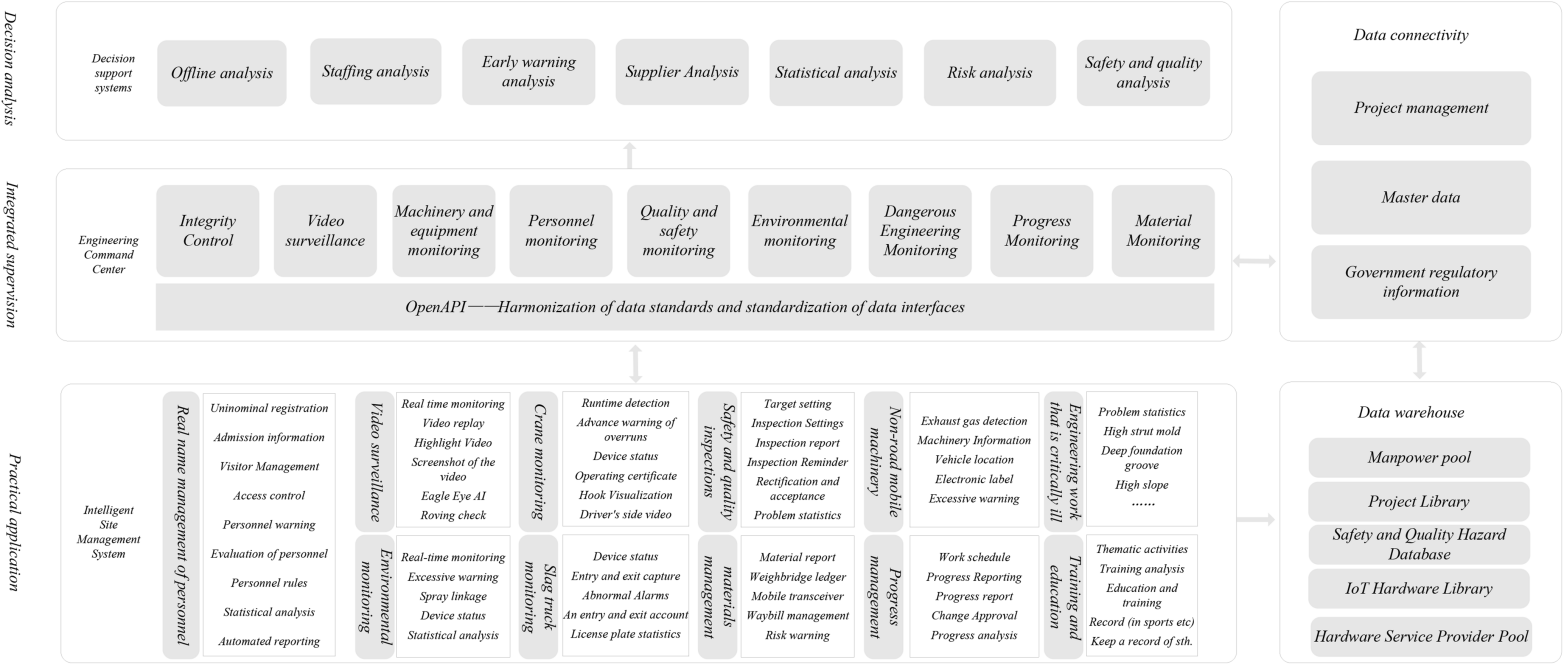
Structural Drawing of Intelligent System of YS Commercial Complex Project.

**Fig 7:**
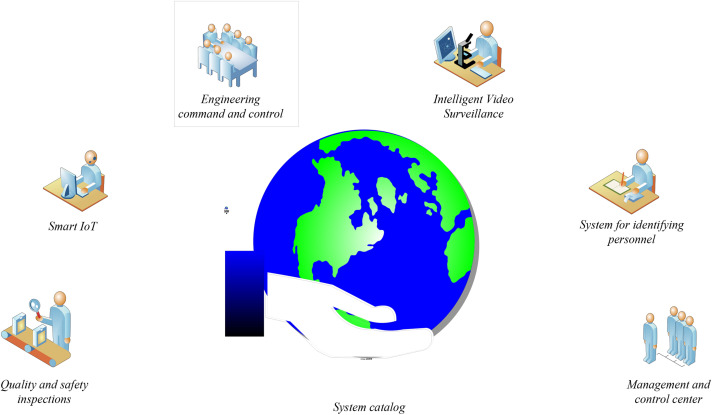
Module division of YS Commercial Complex Project.

To address the vagueness of the intelligent system, its technical architecture is detailed below, including data collection, processing, and application modules (Figs 6 and 7).

In order to facilitate the management of the intelligent system,the intelligent system is divided into a total of six modules,namely,quality and safety inspection,intelligent Internet of things,engineering command and dispatch,intelligent video monitoring,personnel real-name system and control center. As shown in the Fig 7.

#### 4.8.1 Data collection layer.

Data types: (1) Cost data (real-time material prices from suppliers, labor hour records, machinery leasing costs); (2) Functional performance data (HVAC temperature/humidity sensor data, electrical load data, user satisfaction scores); (3) Construction progress data (on-site camera footage, RFID-tagged material delivery records).

Collection methods: (1) Hardware: IoT sensors (temperature, humidity, current), RFID readers, high-definition cameras, mobile terminals (for on-site personnel to input data); (2) Software: API integrations with supplier management systems(to obtain real-time material prices), BIM software (to extract component quantity data), enterprise resource planning (ERP) systems (to integrate financial data).

#### 4.8.2 Data processing layer.

Core algorithms: (1) FAHP model (for calculating functional/cost coefficients, as detailed in Section 2.2); (2) Machine learning models (e.g., LSTM neural network for predicting material price fluctuations, random forest for identifying cost overrun risks); (3) Statistical analysis tools (for calculating cost variances and generating reports).

Data processing workflow: (1) Data cleaning (removing missing or abnormal values); (2) Data integration (combining cost, performance, and progress data into a unified database); (3) Data analysis (applying FAHP and machine learning models to generate insights).

#### 4.8.3 Application modules (Fig 7).

Quality and safety inspection module: Uses computer vision (from on-site cameras) to detect construction quality defects (e.g., concrete cracks) and safety hazards (e.g., unprotected edges), reducing rework costs.

Intelligent IoT module: Monitors real-time data from sensors (e.g., HVAC energy consumption, electrical load) to identify inefficient operation (e.g., excessive cooling in unoccupied areas) and trigger alerts.

Engineering command and dispatch module: Integrates progress and resource data to optimize labor and machinery allocation (e.g., adjusting the number of workers based on task progress) and avoid idle resource costs.

Intelligent video monitoring module: Provides real-time visual monitoring of construction sites, supporting remote supervision and reducing on-site management costs.

Personnel real-name system module: Records labor hours and task completion via RFID or mobile terminals, ensuring accurate labor cost calculation and preventing wage fraud.

Control center module: Serves as the core hub, integrating data from all modules to generate real-time cost dashboards, value coefficient updates, and optimization recommendations. For example, if the control center detects that the cost of Y21 exceeds the target, it automatically recommends prefabrication measures based on historical data.

#### 4.8.4 Real-time monitoring mechanism.

The intelligent system enables real-time cost control by: (1) Updating cost coefficients hourly based on actual expenditure data; (2) Recalculating value coefficients daily to identify emerging low-value indicators; (3) Sending real-time alerts to the project team when costs exceed thresholds (e.g., a 10% increase in material costs for Y21 triggers a recommendation to switch suppliers). This differs from one-time design-phase optimization, as it supports dynamic adjustments throughout the construction process.

### 4.9 Effectiveness verification and comparative baseline

#### 4.9.1 Comparative baseline definition.

The “traditional method” refers to the industry-standard cost management approach for commercial complex projects, which includes: (1) Static budgeting based on initial design documents; (2) Post-hoc cost accounting (no real-time monitoring); (3) VE analysis without FAHP (relying on subjective expert judgment); (4) No intelligent data integration (relying on manual data entry and paper documents). The original project cost (74,886,333.3 yuan) is a well-justified budget based on this traditional method, approved by the project’s investor and in line with local construction cost standards for similar projects (e.g., the average cost of commercial complexes in Sichuan Province in 2023 was 1,500–1,800 yuan/m², and the YS project’s original cost of ~1,466 yuan/m² is within this range).

#### 4.9.2 Optimization results.

The optimized cost achieved through the VE-FAHP-intelligent system framework amounts to 72,740,121.5 yuan, a 2.87% reduction. Breakdown by engineering area:

Civil engineering: Original cost 15,353,662.50 yuan → Optimized cost 14,553,129.90 yuan (reduction: 800,532.60 yuan). This is attributed to material optimization (e.g., high-strength concrete) and process improvement (e.g., prefabricated foundations) recommended by the intelligent system.

Building construction: Original cost 47,577,310.20 yuan → Optimized cost 46,488,133.65 yuan (reduction: 1,089,176.55 yuan). Key measures include decorative material replacement and simplified details, as identified by low value coefficients.

HVAC engineering: Original cost 6,467,977.85 yuan → Optimized cost 6,286,394.50 yuan (reduction: 181,583.35 yuan). Achieved via system design optimization (e.g., variable frequency fans) and intelligent energy management (e.g., adjusting cooling based on occupancy).

Electrical engineering: Original cost 5,487,382.75 yuan → Optimized cost 5,412,463.45 yuan (reduction: 74,919.30 yuan). Driven by LED lighting adoption and intelligent dimming systems.

#### 4.9.3 Significance of cost reduction.

A 2.87% cost reduction holds great significance in the construction industry, where the average profit margin generally ranges from 3% to 5%. The 2,146,211.80 yuan savings from the YS project not only improves the project’s profit margin by ~0.6 percentage points but also enhances its market competitiveness. For example, the savings can be reinvested in value-adding features (e.g., intelligent parking systems) to attract more tenants, creating long-term revenue growth.

## 5 Conclusions

### 5.1 Key findings

VE-FAHP Framework Effectiveness: Integrating VE (with FAST) and FAHP provides a systematic method for cost control in commercial complex projects. By decomposing functions via FAST and quantifying weights via FAHP, the framework accurately identifies low-value indicators (e.g., main construction Y21, V = 0.3917) and prioritizes optimization.

Intelligent System Enhances Real-Time Control: The intelligent system, with its data collection (IoT sensors, API integration), processing (FAHP, machine learning), and application modules, enables dynamic cost monitoring and timely optimization, addressing the limitations of traditional static management.

Significant Cost Reduction: The framework achieves a 2.87% cost reduction (2,146,211.80 yuan) in the YS project, improving profit margins and demonstrating practical value for commercial complex cost management.

### 5.2 Research contributions

Theoretical Contribution: This study integrates FAST into VE and combines it with FAHP, filling the gap in standardized functional analysis and quantitative decision-making in traditional VE. It also enriches the theory of intelligent cost management by detailing the technical architecture of the intelligent system.

Practical Contribution: The framework provides an actionable tool for construction enterprises, with clear steps for selecting VE objects, calculating coefficients, and implementing optimization measures. The intelligent system module design offers a reference for enterprises to digitize cost management.

### 5.3 Limitations

Single Case Study: The framework is validated only in the YS project; future research should test it across multiple projects (e.g., different regions, scales) to enhance generalizability.

Expert Judgment Dependence: FAHP relies on expert pairwise comparisons, which may introduce subjective biases. Future work could integrate more objective data (e.g., historical project performance) to reduce subjectivity.

Intelligent System Scalability: The current system is tailored to the YS project; adapting it to other project types (e.g., residential complexes) requires further customization.

### 5.4 Future research directions

Integrate BIM and Real-Time Data: Combine the VE-FAHP framework with BIM to enable 3D visualization of cost and function data, and integrate real-time data from construction sites (e.g., drone surveys) to improve data accuracy.

Advance Machine Learning Applications: Develop more advanced machine learning models (e.g., deep learning for cost overrun prediction) to enhance the intelligent system’s predictive capabilities.

Expand to Full-Life Cycle Management: Extend the framework to the operation and maintenance phase of commercial complexes, optimizing long-term costs (e.g., energy consumption, maintenance expenses) and maximizing project lifecycle value.

## Supporting information

S1 File Minimal-data-set-definition-2026.01.21. (XLSX)
