# Supplementary material for: Intelligent system in the cost control of commercial complex projects: Data-driven optimization method
Source: PLoS One. 2026 Jun 18;21(6):e0343158. doi: 10.1371/journal.pone.0343158 (PMC13278410; doi:10.1371/journal.pone.0343158)
Supplement: Attachment — Submitted filename: Response to Reviewers.docx [file pone.0343158.s002.docx]

PONE-D-25-38715

Intelligent system in the cost control of commercial complex projects: data-driven optimization method

PLOS ONE
Dear Academic Editor and Reviewers,
 Thank you very much for your careful review of our manuscript and for providing valuable comments and suggestions. These opinions have played a crucial role in helping us improve the quality and academic rigor of the paper. We have carefully studied each comment, made corresponding revisions to the manuscript, and now submit the revised version along with this rebuttal letter to explain our revision details.

Note: In the response letter, all responses are displayed in black font, all modifications and additions are marked in red, and deleted content is indicated in the comments section.

Wishing you a smooth workday and a fulfilling life!

***Response to the Journal Requirements***

**Comment No. 1:** Please ensure that your manuscript meets PLOS ONE's style requirements, including those for file naming. The PLOS ONE style templates can be found at https://journals.plos.org/plosone/s/file?id=wjVg/PLOSOne_formatting_sample_main_body.pdf and https://journals.plos.org/plosone/s/file?id=ba62/PLOSOne_formatting_sample_title_authors_affiliations.pdf.

***Response*:**

We have ensured that your manuscript complies with PLOS ONE's style requirements, including file naming conventions.

**Comment No. 2:** Thank you for stating the following financial disclosure:

[[1] 2025 Liaoning Province Economic and Social Development Research Project (2025lslybwzzkt-141);

[2] General Project of Liaoning Provincial Social Science Planning Fund in 2023: Research on Spatial Overflow and Its Driving Mechanism of Green Technology Innovation Efficiency in Liaoning Construction Industry, L23BGL021

[3] Basic Scientific Research Project of Liaoning Provincial Department of Education in 2023 (Face Project): Research on the Mechanism of the Impact of Formal and Informal Network Interaction on the Safety Behavior of Coal Miners, JYTMS20230827].

Please state what role the funders took in the study. If the funders had no role, please state: "The funders had no role in study design, data collection and analysis, decision to publish, or preparation of the manuscript."

If this statement is not correct you must amend it as needed.

Please include this amended Role of Funder statement in your cover letter; we will change the online submission form on your behalf.

***Response*:**

We wish to amend our financial disclosure to read as follows:

[1]2025 Basic Research Project of the Liaoning Provincial Department of Education: Study on the Impact, Mechanisms, and Countermeasures of Digital Transformation on Sustainable Development Performance in the Construction Industry (LJ112510147007)；

[2] 2025 Liaoning Provincial Economic and Social Development Research Project: The Impact of Digital Innovation Capabilities on the Digital Transformation Performance of Construction Enterprises (2025lslkt-308).

The funder did not have any additional role in the study design, data collection and analysis, decision to publish, or preparation of the manuscript.

This is our revised financial disclosure, and we have incorporated the updated statement into your cover letter.

**Comment No. 3:** We note that your Data Availability Statement is currently as follows: [All relevant data are within the manuscript and its Supporting Information files.]

Please confirm at this time whether or not your submission contains all raw data required to replicate the results of your study. Authors must share the “minimal data set” for their submission. PLOS defines the minimal data set to consist of the data required to replicate all study findings reported in the article, as well as related metadata and methods (https://journals.plos.org/plosone/s/data-availability#loc-minimal-data-set-definition).

For example, authors should submit the following data:

- The values behind the means, standard deviations and other measures reported;

- The values used to build graphs;

- The points extracted from images for analysis.

Authors do not need to submit their entire data set if only a portion of the data was used in the reported study.

If your submission does not contain these data, please either upload them as Supporting Information files or deposit them to a stable, public repository and provide us with the relevant URLs, DOIs, or accession numbers. For a list of recommended repositories, please see https://journals.plos.org/plosone/s/recommended-repositories.

If there are ethical or legal restrictions on sharing a de-identified data set, please explain them in detail (e.g., data contain potentially sensitive information, data are owned by a third-party organization, etc.) and who has imposed them (e.g., an ethics committee). Please also provide contact information for a data access committee, ethics committee, or other institutional body to which data requests may be sent. If data are owned by a third party, please indicate how others may request data access.

***Response*:**

Only a portion of the data was utilized in our report's research.

**Comment No. 4:** PLOS requires an ORCID iD for the corresponding author in Editorial Manager on papers submitted after December 6th, 2016. Please ensure that you have an ORCID iD and that it is validated in Editorial Manager. To do this, go to ‘Update my Information’ (in the upper left-hand corner of the main menu), and click on the Fetch/Validate link next to the ORCID field. This will take you to the ORCID site and allow you to create a new iD or authenticate a pre-existing iD in Editorial Manager.

***Response*:**

We have ensured that we possess an ORCID iD and have verified it within Editorial Manager.

**Comment No. 5:** Please ensure that you include a title page within your main document. You should list all authors and all affiliations as per our author instructions and clearly indicate the corresponding author.

***Response*:**

We have ensured that the title page is included in the main document. We have listed all authors and affiliations according to your journal's author instructions, clearly identifying the corresponding author.

**Comment No. 6:** Thank you for stating the following in the Competing Interests section:

[The authors have declared that no competing interests exist.].

We note that one or more of the authors are employed by a commercial company: Bank of Fuxing.

1. Please provide an amended Funding Statement declaring this commercial affiliation, as well as a statement regarding the Role of Funders in your study. If the funding organization did not play a role in the study design, data collection and analysis, decision to publish, or preparation of the manuscript and only provided financial support in the form of authors' salaries and/or research materials, please review your statements relating to the author contributions, and ensure you have specifically and accurately indicated the role(s) that these authors had in your study. You can update author roles in the Author Contributions section of the online submission form.

Please also include the following statement within your amended Funding Statement.

“The funder provided support in the form of salaries for authors [insert relevant initials], but did not have any additional role in the study design, data collection and analysis, decision to publish, or preparation of the manuscript. The specific roles of these authors are articulated in the ‘author contributions’ section.”

If your commercial affiliation did play a role in your study, please state and explain this role within your updated Funding Statement.

2. Please also provide an updated Competing Interests Statement declaring this commercial affiliation along with any other relevant declarations relating to employment, consultancy, patents, products in development, or marketed products, etc.

Within your Competing Interests Statement, please confirm that this commercial affiliation does not alter your adherence to all PLOS ONE policies on sharing data and materials by including the following statement: "This does not alter our adherence to PLOS ONE policies on sharing data and materials.” (as detailed online in our guide for authors http://journals.plos.org/plosone/s/competing-interests) . If this adherence statement is not accurate and there are restrictions on sharing of data and/or materials, please state these. Please note that we cannot proceed with consideration of your article until this information has been declared.

Please include both an updated Funding Statement and Competing Interests Statement in your cover letter. We will change the online submission form on your behalf.

***Response*:**

Fuxin Bank did not contribute to the research design, data collection and analysis, publication decisions, or manuscript preparation. It is solely the workplace of the fourth author, Li Xiao.

This does not alter our adherence to PLOS ONE policies on sharing data and materials.

**Comment No. 7:** Please amend the manuscript submission data (via Edit Submission) to include author Yifei Li.

***Response*:**

Modified.

**Comment No. 8:**Please amend your authorship list in your manuscript file to include author Li-5306 Li.

If the reviewer comments include a recommendation to cite specific previously published works, please review and evaluate these publications to determine whether they are relevant and should be cited. There is no requirement to cite these works unless the editor has indicated otherwise.

***Response*:**

Modified.

***Response to the comments of Reviewer 1***

**Comment No. 1:** In addition ,the FAST (Function Analysis System Technique), is known VE framework. I suggest to revise the method in the article and align it with FAST.

***Response*:**

We fully agree with this suggestion. To integrate the FAST framework into our Value Engineering (VE) methodology, we have made the following revisions:

First，explicitly introduced FAST as a core component of VE functional analysis in Section 1.2 (Construction Cost Control Modeling Framework) and Section 2.2 (Hierarchical Structure Model Construction). We clarified that FAST provides a standardized framework for decomposing project functions into hierarchical sub-functions (e.g., “support building structure” → “bear vertical loads” → “ensure foundation stability”), addressing the lack of systematic functional analysis in traditional VE.

Second，linked FAST to the hierarchical structure model of the YS project (Section 3.3.1), specifying that the indicator layer (e.g., foundation work, main structure) was derived from FAST functional decomposition. This ensures that our functional analysis is structured, transparent, and aligned with industry-recognized VE practices.

**Comment No. 2:** The introduction should not talk about the case but rather the VE applications in cost reduction.

***Response*:**

We have revised the Introduction section to remove all references to the YS case study and refocus on the application of VE in construction cost reduction. Specifically:

First，added a dedicated paragraph (fourth paragraph of Introduction) reviewing VE’s role in cost reduction, citing key studies such as Ibusuki and Kaminski (2007) (VE-target costing integration in automotive industry), Talebnia et al. (2017) (VE-target costing link for profit improvement), and Latif et al. (2020) (VE application in sustainable energy infrastructure). This contextualizes VE’s relevance to construction cost control.

Second，removed preliminary mentions of the YS project (originally in the “traditional cost management limitations” paragraph) and reserved all case-specific content for Section 3 (Cost Control Application and Effectiveness of YS Project), ensuring the Introduction maintains a broad, literature-driven focus.

**Comment No. 3:** Authors need to identify possible drawback on the traditional VE and how the Fuzzy AHP system solve it

***Response*:**

We have explicitly addressed the limitations of traditional VE and the mitigating role of Fuzzy Analytic Hierarchy Process (FAHP) in the revised Introduction (third paragraph) and Section 1.2:

First，drawbacks of traditional VE:

Heavy reliance on subjective expert judgment, leading to ambiguous evaluations of functional importance.

Lack of a standardized framework for decomposing complex project functions, resulting in imprecise cost allocation.

Inability to quantify fuzziness in multi-criteria decision-making (e.g., “which sub-function is more critical for user satisfaction”).

Second，how FAHP solves these issues:

Introduces fuzzy logic to convert qualitative expert opinions into quantitative data, reducing ambiguity (e.g., using 1–9 scales to quantify “slightly more important” or “extremely important”).

Structures complex decision problems into a hierarchical model (target → criterion → indicator), enabling systematic analysis of functional and cost factors.

Includes a rigorous consistency test (CR < 0.1) to validate the reliability of expert judgments, avoiding arbitrary decision-making (detailed in Section 2.2.4).

Special thanks to you for your review and comments.

***Response to the comments of Reviewer 2***

**Comment No. 1:** The "Intelligent System" is Vague and Underdeveloped: The title and abstract heavily promote an "intelligent system," but this is the paper's weakest point. The system is described only in a brief, non-technical section (Figs. 6 & 7) that lists generic module names (e.g., "Smart IoT," "Engineering command and control") without explaining:

What data is collected? (e.g., real-time material costs, labor hours, sensor data?)

How is it collected? (What hardware/software is used?)

What algorithms constitute the "intelligence"? Is it just the FAHP model, or is there machine learning for prediction?

How does it enable "real-time monitoring"? The paper describes a one-time design-phase optimization, not real-time control.

Suggestion: Either substantially develop the technical architecture of the intelligent system with specific technologies and data flows, or reframe the paper's contribution around the integrated VE-FAHP framework itself, which is robustly demonstrated.

***Response*:**

We have substantially expanded the technical details of the intelligent system in a new subsection (Section 3.8: Technical Architecture of the Intelligent System), addressing all gaps raised:

First，data collection:

Specified data types: Real-time material prices, labor hour records, HVAC sensor data (temperature/humidity), electrical load data, and construction progress data (RFID-tagged materials, on-site camera footage).

Described collection methods: IoT sensors (temperature, current), RFID readers, high-definition cameras, and API integration with supplier management systems/BIM/ERP software.

Second，data processing:

Identified core algorithms: FAHP (for coefficient calculation), LSTM neural networks (material price prediction), and random forests (cost overrun risk detection).

Outlined workflow: Data cleaning → integration → analysis (e.g., real-time coefficient recalculation).

Third，“intelligence” and real-time monitoring:

Clarified that “intelligence” combines FAHP (quantitative decision-making) and machine learning (predictive analytics).

Explained real-time monitoring: Hourly cost coefficient updates, daily value coefficient recalculations, and automated alerts for cost overruns (e.g., a 10% increase in main construction costs triggers supplier switch recommendations).

Fourth，module details: Expanded on Figure 7 modules (e.g., “Intelligent IoT module” monitors energy consumption to optimize HVAC operation; “Personnel real-name system” prevents wage fraud via RFID labor tracking).

**Comment No. 2:** Lack of a Clear Comparative Baseline: The paper claims the model outperforms "traditional methods," but it does not define what these traditional methods are. Was the original project cost (74.8 million yuan) a result of poor traditional management, or was it a well-justified budget? The 2.87% saving is presented as a success, but without context:

Suggestion: Compare the optimized cost not just to the original budget, but to a proper baseline. This could be the cost estimated by standard industry practices or a detailed explanation of why the original design was inefficient. Justify that the saving is significant in the context of construction project margins.

Inconsistent Terminology and Currency: The text and tables inconsistently use "yuan" and the dollar symbol "$". For example, Table 13 uses "yuan" but the results section on pages 41-42 uses "$". This is confusing and unprofessional.

Suggestion: Consistently use the correct currency symbol ("¥" for yuan) throughout the manuscript.

***Response*:**

We have addressed both issues comprehensively:

First，comparative baseline:

Defined “traditional methods” as industry-standard practices: Static budgeting, post-hoc accounting, VE without FAHP (subjective judgment), and manual data entry (Section 3.9.1).

Justified the original cost (¥74,886,333.3) as a well-approved budget aligned with local standards (average cost of Sichuan commercial complexes: ¥1,500–1,800/m²; YS project: ~¥1,466/m²).

Explained the significance of 2.87% savings: Construction industry profit margins are typically 3–5%, so ¥2.15M savings improves margins by ~0.6 percentage points and enables reinvestment in value-adding features.

Second，currency consistency:

Replaced all “$” symbols with “¥” (yuan) throughout the manuscript, including tables (e.g., Table 13, 14) and results sections (Pages 41–42).

**Comment No. 3:** The abstract should be more precise. Instead of "employing advanced software and hardware," specify the core methodology: "by integrating Value Engineering with a Fuzzy AHP model." The introduction's literature review is adequate but could be more focused on intelligent systems in construction management to better set up the paper's claimed contribution.

***Response*:**

We have revised the Abstract and Introduction accordingly:

First，abstract:

Replaced “employing advanced software and hardware” with “by integrating Value Engineering with a Fuzzy AHP model and an intelligent system (IoT sensors, machine learning algorithms).”

Clarified the core methodology and outcomes (2.87% cost reduction) more explicitly.

Second，introduction literature review:

Added references to intelligent systems in construction, such as Stergiou and Psannis (2022) (digital twin for IoT big data management) and Ochella et al. (2022) (AI for engineering prognostics).

Linked these studies to our gap: Few works integrate intelligent systems with VE-FAHP for commercial complex cost control.

**Comment No. 4:** The results and discussion section is the core of the paper but is poorly structured. It mixes methodology (how coefficients are calculated) with results (the final numbers) and discussion (why a value is high or low). Present the calculation of FAHP weights, functional coefficients, cost coefficients, and finally the value coefficients for the YS case study. This is the "how." Clearly present the key result—Table 14 showing the value coefficients and identifying components with V<1. The discussion should analyze why certain components (like main construction Y21) have a low V. Discuss the practical optimization measures taken (e.g., "product selection optimization, installation process improvement" mentioned on page 42) in detail here, linking them back to the model's findings. This is the "so what."

***Response*:**

We have restructured Section 3 to separate methodology, results, and discussion clearly:

First，methodology (how): Concentrated in Subsections 3.1–3.7 (object selection, VE team establishment, coefficient calculation), with detailed FAHP steps (e.g., intermediate weight calculations in Section 2.2.3).

Second，results (what): Presented in Table 14 (value coefficients) and Section 3.9.2 (optimization results: cost reductions by engineering area).

Third，discussion (so what): Added a dedicated subsection (3.7: Program Improvement and Optimization) analyzing low-value indicators (e.g., Y21, V=0.3917) and linking optimization measures to model findings:

Main construction (Y21): High-strength concrete reduced material dosage by 15%; prefabrication cut labor costs by 12%.

Decoration (Y24): Domestic tile replacement reduced material costs by 25%; simplified ceiling patterns cut construction costs by 18%.

**Comment No. 5:** The conclusion is very brief and simply restates the results. It should be strengthened to: (1) Summarize the key findings; (2) Explicitly state the research contribution (e.g., "This study demonstrates a structured framework for prioritizing cost optimization in complex projects."); (3) Acknowledge the study's limitations more thoroughly (e.g., the model relies on expert judgment for FAHP, the case study is a single project, the "intelligent system" aspect is conceptual); (4) Provide concrete directions for future research (e.g., integrating real-time BIM data, applying machine learning to dynamically update value coefficients, testing the framework on multiple project types).

***Response*:**

We have expanded the Conclusion into four structured subsections:

First，key Findings: Summarized VE-FAHP effectiveness, intelligent system value, and 2.87% cost reduction.

Second，research Contributions:

Theoretical: Integrated FAST into VE; enriched intelligent cost management theory.

Practical: Provided actionable steps for enterprises; module design for digitization.

Third，limitations:

Single case study (YS project); limited generalizability.

FAHP relies on expert judgment; potential subjectivity.

Intelligent system tailored to YS; scalability to other project types needs testing.

Fourth，future Directions:

Integrate BIM and drone survey data for 3D visualization.

Develop deep learning models for cost overrun prediction.

Extend the framework to operation/maintenance phases.

**Comment No. 6:** The FAHP calculation process in Sections 2.2 and 3.3.3 is confusing. For example, in the calculation of the criterion layer weights (page 31), the formulas are shown but the intermediate steps for normalizing the judgment matrix are missing, making it difficult to verify the results.

***Response*:**

We have added detailed intermediate calculations for FAHP in Section 2.2.3 (Calculate Weights) using the civil engineering indicator layer (Table 8) as an example:

First，row-wise multiplication: $W_{Y_{11}}=1\text{×}\text{2}\text{×}\text{2}\text{=}\text{4}$;$W_{Y_{12}}=\text{1}/2\text{×}\text{1}\text{×}\text{2}\text{=}\text{1}$;$W_{Y_{13}}=\text{1}/2\text{×}\text{1}/2\text{×}\text{1}\text{=}\text{0.25}$.

Second，nth root extraction:$\bar{\text{W}_{\text{Y}_{\text{11}}}}=\sqrt[3]{4}\text{≈}\text{1.587}$ ;$\bar{\text{W}_{\text{Y}_{\text{1}\text{2}}}}=1$;$\bar{\text{W}_{\text{Y}_{\text{1}\text{3}}}}\text{≈}\text{0.63}$.

Third，normalization:$sum=\text{3.217}$ ;$\bar{\text{W}_{\text{Y}_{\text{1}\text{1}}}}=\text{1.587}/\text{3.217}\text{≈}\text{0.49}$;$\bar{\text{W}_{\text{Y}_{\text{1}\text{2}}}}\text{≈}\text{0.31}$;$\bar{\text{W}_{\text{Y}_{\text{1}\text{3}}}}\text{≈}\text{0.20}$.

This step-by-step breakdown ensures transparency and verifiability of FAHP results.

**Comment No. 7:** Figures 1, 6, and 7 are rudimentary and lack professional quality. Figure 1's data source ("AiMedia Consulting") needs a proper citation. Figures 6 and 7 need to be properly labeled and integrated into the text.

***Response*:**

We have addressed the figure-related issues:

First，figure 1 (Retail consumption market size): Added a formal citation to AiMedia Consulting (2023) in the figure caption and Introduction (first paragraph: “According to AiMedia Consulting (2023), the retail consumption industry…”).

Second，figures 6 and 7: Enhanced their integration into the text by:

Adding detailed captions explaining the intelligent system structure (Figure 6: “Structural Drawing of Intelligent System for YS Commercial Complex Project, including data collection, processing, and application layers”).

Referencing specific modules in the text (e.g., Section 3.8 links Figure 7’s “Intelligent IoT module” to HVAC energy optimization).

**Comment No. 8:** The manuscript requires thorough proofreading by a native English speaker or professional editing service to correct grammatical errors and improve sentence fluency (e.g., "commercial complicated project" should be "commercial complex project").

***Response*:**

The entire manuscript has been proofread by a native English speaker with expertise in construction engineering academia. Key revisions include:

Correcting “commercial complicated project” to “commercial complex project” (consistently applied).

Fixing subject-verb agreement errors (e.g., “The team aim” → “The team aims”).

Improving sentence fluency (e.g., rephrasing “the utilisation of intelligent technology in cost decision-making is insufficient” to “intelligent technology is underutilized in cost decision-making”).

Once again, we thank the editor and reviewers for their time and insights. We believe these revisions have significantly strengthened the manuscript’s scientific rigor, clarity, and contribution to the field. We hope the revised version meets the journal’s publication standards.

Sincerely,

Yifei Li
