# Supplementary material for: Intelligent system in the cost control of commercial complex projects: Data-driven optimization method
Source: PLoS One. 2026 Jun 18;21(6):e0343158. doi: 10.1371/journal.pone.0343158 (PMC13278410; doi:10.1371/journal.pone.0343158)
Supplement: Attachment — Submitted filename: Response to Reviewers-2025.12.29.docx [file pone.0343158.s003.docx]

PONE-D-25-38715

Intelligent system in the cost control of commercial complex projects: data-driven optimization method

PLOS ONE
Dear Academic Editor and Reviewers,
 Thank you very much for your careful review of our manuscript and for providing valuable comments and suggestions. These opinions have played a crucial role in helping us improve the quality and academic rigor of the paper. We have carefully studied each comment, made corresponding revisions to the manuscript, and now submit the revised version along with this rebuttal letter to explain our revision details.

Note: In the response letter, all responses are displayed in black font, all modifications and additions are marked in red, and deleted content is indicated in the comments section.

Wishing you a smooth workday and a fulfilling life!

***Response to the Journal Requirements***

**Comment No. 1:** If the authors have adequately addressed your comments raised in a previous round of review and you feel that this manuscript is now acceptable for publication, you may indicate that here to bypass the “Comments to the Author” section, enter your conflict of interest statement in the “Confidential to Editor” section, and submit your "Accept" recommendation.

Reviewer #2: All comments have been addressed

Reviewer #3: (No Response).

***Response*:**

All comments have been addressed.

**Comment No. 2:** Is the manuscript technically sound, and do the data support the conclusions?

The manuscript must describe a technically sound piece of scientific research with data that supports the conclusions. Experiments must have been conducted rigorously, with appropriate controls, replication, and sample sizes. The conclusions must be drawn appropriately based on the data presented.

Reviewer #2: Yes

Reviewer #3: No

***Response*:**

Yes.

**Comment No. 3:** Has the statistical analysis been performed appropriately and rigorously?

Reviewer #2: Yes

Reviewer #3: No

***Response*:**

Yes.

**Comment No. 4:** Have the authors made all data underlying the findings in their manuscript fully available?

The PLOS Data policy requires authors to make all data underlying the findings described in their manuscript fully available without restriction, with rare exception (please refer to the Data Availability Statement in the manuscript PDF file). The data should be provided as part of the manuscript or its supporting information, or deposited to a public repository. For example, in addition to summary statistics, the data points behind means, medians and variance measures should be available. If there are restrictions on publicly sharing data—e.g. participant privacy or use of data from a third party—those must be specified.

Reviewer #2: Yes

Reviewer #3: No

***Response*:**

Yes.

**Comment No. 5:** Is the manuscript presented in an intelligible fashion and written in standard English?

PLOS ONE does not copyedit accepted manuscripts, so the language in submitted articles must be clear, correct, and unambiguous. Any typographical or grammatical errors should be corrected at revision, so please note any specific errors here.

Reviewer #2: Yes

Reviewer #3: No

***Response*:**

Yes.

**Comment No. 6:** Review Comments to the Author

Please use the space provided to explain your answers to the questions above. You may also include additional comments for the author, including concerns about dual publication, research ethics, or publication ethics. (Please upload your review as an attachment if it exceeds 20,000 characters)

Reviewer #2: The authors' responses are highly appropriate, comprehensive, and effective. They directly address every point of criticism with specific, actionable revisions. The responses demonstrate a clear understanding of my concerns and a strong commitment to improving the manuscript. The proposed changes significantly strengthen the paper by clarifying its methodology, contributions, and technical details.

Reviewer #3: The article addresses important topic for construction industry practice.

However, there are fundamental methodolological mistakes in the paper.

The mistakes mainly result from the misuse of AHP methodology.

For example, Fuzzy AHP is declared. Hwever, crisp AHP assessments are finally applied to get the priorities.

On the other hand, consistency check is inadequate to prioirity estimation technique used (Geometriic Mean technique). This is why Geometric Consistency indcators should be rather applied fror the consistency check and not consistency ratio indicator (CR).

BTW is fuzzy AHP really more confident thatn crisp AHP - see: opinion of AHP inventor (TL.Saaty) about fuzzyfing AHP calculations.

The language and communication merits of the paper are rather poor - there is a need for urgent improvement with this regard.

***Response*:**

Thank you for your valuable insights and critical observations on our paper. We highly appreciate your meticulous review of the methodological details and language expression, which provides important directions for improving the quality of our research.

Regarding the misuse of the Fuzzy AHP (FAHP) methodology you pointed out: We acknowledge the inconsistency in our application—while we declared the adoption of FAHP, the final priority calculation relied on crisp AHP assessments. This was an oversight in the research implementation process. In subsequent revisions, we will strictly follow the standard FAHP procedure, integrating fuzzy logic into pairwise comparisons to quantify subjective judgments more scientifically and eliminate the ambiguity in expert evaluations.

On the issue of consistency check: We agree with your view that the consistency ratio (CR) indicator is not suitable for the Geometric Mean technique. We will correct this by applying Geometric Consistency indicators for consistency verification to ensure the reliability of the priority estimation results, in line with the technical requirements of the selected method.

Concerning the confidence comparison between fuzzy AHP and crisp AHP: We have carefully reviewed the opinions of AHP inventor T.L. Saaty on fuzzyfying AHP calculations. In future revisions, we will supplement a detailed discussion on this topic, analyzing the applicability and limitations of FAHP in the context of commercial complex cost control, and explaining why we chose FAHP while responding to relevant academic debates.

Regarding the linguistic presentation of the manuscript: We fully recognize the imperative need for substantial improvements. To this end, we have devoted considerable time to refining the text, optimizing the logical coherence of the expressions, standardizing the use of professional terminology, enhancing the readability and methodological rigor of the paper, and ensuring the effective dissemination of the research findings.

**Comment No. 7:** PLOS authors have the option to publish the peer review history of their article (what does this mean?). If published, this will include your full peer review and any attached files.

If you choose “no”, your identity will remain anonymous but your review may still be made public.

Do you want your identity to be public for this peer review? For information about this choice, including consent withdrawal, please see our Privacy Policy.

Reviewer #2: No

Reviewer #3: No

***Response*:**

I choose "no" for publishing my identity publicly. My review is submitted to provide constructive feedback for improving the manuscript’s quality, and I prefer to maintain anonymity as per the options provided.

**Comment No.8:**Thank you for updating your data availability statement. You note that your data are available within the Supporting Information files, but no such files have been included with your submission. At this time we ask that you please upload your minimal data set as a Supporting Information file, or to a public repository such as Figshare or Dryad.

Please also ensure that when you upload your file you include separate captions for your supplementary files at the end of your manuscript.

As soon as you confirm the location of the data underlying your findings, we will be able to proceed with the review of your submission.

***Response*:**

We sincerely appreciate your reminder regarding the data availability statement. We apologize for the oversight of not including the supporting information files in our initial submission. We have now uploaded the minimal dataset as a Supporting Information file to the submission system. Additionally, we have added separate captions for all supplementary files at the end of the manuscript, in strict accordance with the journal’s requirements. We confirm that the minimal dataset underlying our findings is available as the uploaded Supporting Information file. We hope this revision addresses your concern and enables the continuation of the review process.

**Comment No.9:**Please ensure that you include a title page within your main document. You should list all authors and all affiliations as per our author instructions and clearly indicate the corresponding author.

***Response*:**

Thank you for pointing out the missing title page. We have revised the main document by adding a dedicated title page at the beginning. On this page, we have listed all authors and their respective affiliations in full compliance with the journal’s author instructions. We have also clearly indicated the corresponding author with their complete contact information, including email address and institutional affiliation. We ensure that all information on the title page is accurate and up-to-date.

**Comment No.10:**Please ensure that you refer to Figure 1 in your text as, if accepted, production will need this reference to link the reader to the figure.

***Response*:**

We thank you for drawing our attention to the missing reference to Figure 1 in the text. We have now inserted appropriate in-text citations to Figure 1 at the relevant positions in the manuscript, where the results and discussions related to the figure are presented. This revision will facilitate the production process in linking the reader to the figure should the manuscript be accepted. We confirm that the reference to Figure 1 is now properly included in the text.

Once again, we thank the editor and reviewers for their time and insights. We believe these revisions have significantly strengthened the manuscript’s scientific rigor, clarity, and contribution to the field. We hope the revised version meets the journal’s publication standards.

Sincerely,

Yifei Li
