# Supplementary material for: Intelligent system in the cost control of commercial complex projects: Data-driven optimization method
Source: PLoS One. 2026 Jun 18;21(6):e0343158. doi: 10.1371/journal.pone.0343158 (PMC13278410; doi:10.1371/journal.pone.0343158)
Supplement: Attachment — Submitted filename: Response to Reviewers-2026.01.21.docx [file pone.0343158.s004.docx]

PONE-D-25-38715

Intelligent system in the cost control of commercial complex projects: data-driven optimization method

PLOS ONE
Dear Academic Editor and Reviewers,
 Thank you very much for your careful review of our manuscript and for providing valuable comments and suggestions. These opinions have played a crucial role in helping us improve the quality and academic rigor of the paper. We have carefully studied each comment, made corresponding revisions to the manuscript, and now submit the revised version along with this rebuttal letter to explain our revision details.

Note: In the response letter, all responses are displayed in black font, all modifications and additions are marked in red, and deleted content is indicated in the comments section.

Wishing you a smooth workday and a fulfilling life!

***Response to the Journal Requirements***

**Comment :** There are some typing mistakes which have been found in this paper. Kindly proofreading this manuscript carefully and correct this mistake.

For example: Figure 2, Kindly adjust this phrase word: Vertical channel.

Figure 4, Missing some words at mid block: Preparat, etc.

so on.

***Response*:**Thank you for your careful review and valuable comments on our manuscript. We have thoroughly proofread the entire paper and corrected the identified typos and deficiencies as follows:

1.Regarding Figure 2, the phrase "Vertical channel" has been adjusted to "Logical Analysis of the Necessity of Applying Value Engineering" to align with the actual theme of the figure.

2.For Figure 4, the incomplete word "Preparat" has been supplemented to "Preparation", completing the workflow as "Define the target group → Form a team → Make a plan → Data collection → Functional analysis → Functional evaluation → Determination of target costs → Program selection, material procurement, equipment selection → Preparation".

3.We have standardized the subscript formats (e.g., Y₁, Y₁₁, Y₂₁) and adjusted the layout of formulas (1)-(8) to ensure clarity and readability.

4.Inconsistencies in table numbering and unit expressions (e.g., "yuan" formatting) have been rectified, and redundant commas and missing punctuation marks have been corrected to comply with academic writing norms.

Additionally, we have polished the language of the manuscript to enhance its fluency and professionalism. For example, optimizing adjective collocations and adjusting sentence structures to make the expression more precise and logical.

All revisions have been made carefully without altering the original research content and conclusions. We hope the revised manuscript meets the publication requirements. Thank you again for your valuable suggestions, which have helped improve the quality of our paper significantly.

Sincerely,

Yifei Li
