# Supplementary material for: Intelligent system in the cost control of commercial complex projects: Data-driven optimization method
Source: PLoS One. 2026 Jun 18;21(6):e0343158. doi: 10.1371/journal.pone.0343158 (PMC13278410; doi:10.1371/journal.pone.0343158)
Supplement: Peer review history

# Peer review: Decision Letter 0

**DOI:** 10.1371/journal.pone.0343158.r001

**Authors:** Tien Anh Tran

26 Sep 2025

PONE-D-25-38715

Intelligent system in the cost control of commercial complex projects: data-driven optimization method

PLOS ONE

Dear Dr. Li,

Thank you for submitting your manuscript to PLOS ONE. After careful consideration, we feel that it has merit but does not fully meet PLOS ONE’s publication criteria as it currently stands. Therefore, we invite you to submit a revised version of the manuscript that addresses the points raised during the review process.

==============================

**The paper must be addressed all comments to improve the quality of paper. The authors read and revise carefully their paper. I am looking forward to receiving your revised paper.**

==============================

Please submit your revised manuscript by Nov 10 2025 11:59PM. If you will need more time than this to complete your revisions, please reply to this message or contact the journal office at plosone@plos.org. When you're ready to submit your revision, log on to https://www.editorialmanager.com/pone/ and select the 'Submissions Needing Revision' folder to locate your manuscript file.

Please include the following items when submitting your revised manuscript:

- A rebuttal letter that responds to each point raised by the academic editor and reviewer(s). You should upload this letter as a separate file labeled 'Response to Reviewers'.
- A marked-up copy of your manuscript that highlights changes made to the original version. You should upload this as a separate file labeled 'Revised Manuscript with Track Changes'.
- An unmarked version of your revised paper without tracked changes. You should upload this as a separate file labeled 'Manuscript'.

If you would like to make changes to your financial disclosure, please include your updated statement in your cover letter. Guidelines for resubmitting your figure files are available below the reviewer comments at the end of this letter.

If applicable, we recommend that you deposit your laboratory protocols in protocols.io to enhance the reproducibility of your results. Protocols.io assigns your protocol its own identifier (DOI) so that it can be cited independently in the future. For instructions see: https://journals.plos.org/plosone/s/submission-guidelines#loc-laboratory-protocols. Additionally, PLOS ONE offers an option for publishing peer-reviewed Lab Protocol articles, which describe protocols hosted on protocols.io. Read more information on sharing protocols at https://plos.org/protocols?utm_medium=editorial-email&utm_source=authorletters&utm_campaign=protocols.

We look forward to receiving your revised manuscript.

Kind regards,

Tien Anh Tran

Academic Editor

PLOS ONE

Journal Requirements:

When submitting your revision, we need you to address these additional requirements.

1. Please ensure that your manuscript meets PLOS ONE's style requirements, including those for file naming. The PLOS ONE style templates can be found at

https://journals.plos.org/plosone/s/file?id=wjVg/PLOSOne_formatting_sample_main_body.pdf and https://journals.plos.org/plosone/s/file?id=ba62/PLOSOne_formatting_sample_title_authors_affiliations.pdf

2. Thank you for stating the following financial disclosure:

[[1] 2025 Liaoning Province Economic and Social Development Research Project (2025lslybwzzkt-141);

[2] General Project of Liaoning Provincial Social Science Planning Fund in 2023: Research on Spatial Overflow and Its Driving Mechanism of Green Technology Innovation Efficiency in Liaoning Construction Industry, L23BGL021

[3] Basic Scientific Research Project of Liaoning Provincial Department of Education in 2023 (Face Project): Research on the Mechanism of the Impact of Formal and Informal Network Interaction on the Safety Behavior of Coal Miners, JYTMS20230827].

Please state what role the funders took in the study.  If the funders had no role, please state: "The funders had no role in study design, data collection and analysis, decision to publish, or preparation of the manuscript."

If this statement is not correct you must amend it as needed.

Please include this amended Role of Funder statement in your cover letter; we will change the online submission form on your behalf.

3. We note that your Data Availability Statement is currently as follows: [All relevant data are within the manuscript and its Supporting Information files.]

Please confirm at this time whether or not your submission contains all raw data required to replicate the results of your study. Authors must share the “minimal data set” for their submission. PLOS defines the minimal data set to consist of the data required to replicate all study findings reported in the article, as well as related metadata and methods (https://journals.plos.org/plosone/s/data-availability#loc-minimal-data-set-definition).

For example, authors should submit the following data:

- The values behind the means, standard deviations and other measures reported;

- The values used to build graphs;

- The points extracted from images for analysis.

Authors do not need to submit their entire data set if only a portion of the data was used in the reported study.

If your submission does not contain these data, please either upload them as Supporting Information files or deposit them to a stable, public repository and provide us with the relevant URLs, DOIs, or accession numbers. For a list of recommended repositories, please see https://journals.plos.org/plosone/s/recommended-repositories.

If there are ethical or legal restrictions on sharing a de-identified data set, please explain them in detail (e.g., data contain potentially sensitive information, data are owned by a third-party organization, etc.) and who has imposed them (e.g., an ethics committee). Please also provide contact information for a data access committee, ethics committee, or other institutional body to which data requests may be sent. If data are owned by a third party, please indicate how others may request data access.

4. PLOS requires an ORCID iD for the corresponding author in Editorial Manager on papers submitted after December 6th, 2016. Please ensure that you have an ORCID iD and that it is validated in Editorial Manager. To do this, go to ‘Update my Information’ (in the upper left-hand corner of the main menu), and click on the Fetch/Validate link next to the ORCID field. This will take you to the ORCID site and allow you to create a new iD or authenticate a pre-existing iD in Editorial Manager.

5. Please ensure that you include a title page within your main document. You should list all authors and all affiliations as per our author instructions and clearly indicate the corresponding author.

6. Thank you for stating the following in the Competing Interests section:

[The authors have declared that no competing interests exist.].

We note that one or more of the authors are employed by a commercial company: Bank of Fuxing.

1. Please provide an amended Funding Statement declaring this commercial affiliation, as well as a statement regarding the Role of Funders in your study. If the funding organization did not play a role in the study design, data collection and analysis, decision to publish, or preparation of the manuscript and only provided financial support in the form of authors' salaries and/or research materials, please review your statements relating to the author contributions, and ensure you have specifically and accurately indicated the role(s) that these authors had in your study. You can update author roles in the Author Contributions section of the online submission form.

Please also include the following statement within your amended Funding Statement.

“The funder provided support in the form of salaries for authors [insert relevant initials], but did not have any additional role in the study design, data collection and analysis, decision to publish, or preparation of the manuscript. The specific roles of these authors are articulated in the ‘author contributions’ section.”

If your commercial affiliation did play a role in your study, please state and explain this role within your updated Funding Statement.

2. Please also provide an updated Competing Interests Statement declaring this commercial affiliation along with any other relevant declarations relating to employment, consultancy, patents, products in development, or marketed products, etc.

Within your Competing Interests Statement, please confirm that this commercial affiliation does not alter your adherence to all PLOS ONE policies on sharing data and materials by including the following statement: "This does not alter our adherence to  PLOS ONE policies on sharing data and materials.” (as detailed online in our guide for authors http://journals.plos.org/plosone/s/competing-interests) . If this adherence statement is not accurate and there are restrictions on sharing of data and/or materials, please state these. Please note that we cannot proceed with consideration of your article until this information has been declared.

Please include both an updated Funding Statement and Competing Interests Statement in your cover letter. We will change the online submission form on your behalf.

7. Please amend the manuscript submission data (via Edit Submission) to include author Yifei Li.

8. Please amend your authorship list in your manuscript file to include author Li-5306 Li.

If the reviewer comments include a recommendation to cite specific previously published works, please review and evaluate these publications to determine whether they are relevant and should be cited. There is no requirement to cite these works unless the editor has indicated otherwise.

[Note: HTML markup is below. Please do not edit.]

Reviewers' comments:

Reviewer's Responses to Questions

**Comments to the Author**

1. Is the manuscript technically sound, and do the data support the conclusions?

The manuscript must describe a technically sound piece of scientific research with data that supports the conclusions. Experiments must have been conducted rigorously, with appropriate controls, replication, and sample sizes. The conclusions must be drawn appropriately based on the data presented.

Reviewer #1: Yes

Reviewer #2: No

**********

2. Has the statistical analysis been performed appropriately and rigorously?

Reviewer #1: N/A

Reviewer #2: No

**********

3. Have the authors made all data underlying the findings in their manuscript fully available?

The PLOS Data policy requires authors to make all data underlying the findings described in their manuscript fully available without restriction, with rare exception (please refer to the Data Availability Statement in the manuscript PDF file). The data should be provided as part of the manuscript or its supporting information, or deposited to a public repository. For example, in addition to summary statistics, the data points behind means, medians and variance measures should be available. If there are restrictions on publicly sharing data—e.g. participant privacy or use of data from a third party—those must be specified.

Reviewer #1: Yes

Reviewer #2: No

**********

4. Is the manuscript presented in an intelligible fashion and written in standard English?

PLOS ONE does not copyedit accepted manuscripts, so the language in submitted articles must be clear, correct, and unambiguous. Any typographical or grammatical errors should be corrected at revision, so please note any specific errors here.

Reviewer #1: Yes

Reviewer #2: Yes

**********

5. Review Comments to the Author

Please use the space provided to explain your answers to the questions above. You may also include additional comments for the author, including concerns about dual publication, research ethics, or publication ethics. (Please upload your review as an attachment if it exceeds 20,000 characters)

Reviewer #1: The paper may have some merit, about the enhancement of VE with experts fuzzy system. However, there is no clear methodology on how the integration is made.

1. In addition ,the FAST (Function Analysis System Technique), is known VE framework. I suggest to revise the method in the article and align it with FAST.

2. The introduction should not talk about the case but rather the VE applications in cost reduction.

3. Authors need to identify possible drawback on the traditional VE and how the Fuzzy AHP system solve it

Reviewer #2: This paper proposes an intelligent, data-driven cost control system for commercial complex construction projects, integrating Value Engineering (VE) with the Fuzzy Analytic Hierarchy Process (FAHP). The core of the method is a model that calculates functional, cost, and value coefficients for different project components (e.g., civil works, HVAC, electrical). Components with a low value coefficient (V<1) are flagged for optimization, as they represent poor cost-to-function value. The model is applied to a case study, the "YS Commercial Complex" project. The authors claim that by optimizing the design and construction methods of low-value components, the intelligent system reduced the total project cost from 74,886,333.3 yuan to 72,740,121.5 yuan, achieving a 2.87% cost saving compared to traditional methods.

The following feedback is structured from major conceptual issues to specific presentational details:

1. The "Intelligent System" is Vague and Underdeveloped: The title and abstract heavily promote an "intelligent system," but this is the paper's weakest point. The system is described only in a brief, non-technical section (Figs. 6 & 7) that lists generic module names (e.g., "Smart IoT," "Engineering command and control") without explaining:

What data is collected? (e.g., real-time material costs, labor hours, sensor data?)

How is it collected? (What hardware/software is used?)

What algorithms constitute the "intelligence"? Is it just the FAHP model, or is there machine learning for prediction?

How does it enable "real-time monitoring"? The paper describes a one-time design-phase optimization, not real-time control.

Suggestion: Either substantially develop the technical architecture of the intelligent system with specific technologies and data flows, or reframe the paper's contribution around the integrated VE-FAHP framework itself, which is robustly demonstrated.

2. Lack of a Clear Comparative Baseline: The paper claims the model outperforms "traditional methods," but it does not define what these traditional methods are. Was the original project cost (74.8 million yuan) a result of poor traditional management, or was it a well-justified budget? The 2.87% saving is presented as a success, but without context:

Suggestion: Compare the optimized cost not just to the original budget, but to a proper baseline. This could be the cost estimated by standard industry practices or a detailed explanation of why the original design was inefficient. Justify that the saving is significant in the context of construction project margins.

Inconsistent Terminology and Currency: The text and tables inconsistently use "yuan" and the dollar symbol "$". For example, Table 13 uses "yuan" but the results section on pages 41-42 uses "$". This is confusing and unprofessional.

Suggestion: Consistently use the correct currency symbol ("¥" for yuan) throughout the manuscript.

3. The abstract should be more precise. Instead of "employing advanced software and hardware," specify the core methodology: "by integrating Value Engineering with a Fuzzy AHP model." The introduction's literature review is adequate but could be more focused on intelligent systems in construction management to better set up the paper's claimed contribution.

4. The results and discussion section is the core of the paper but is poorly structured. It mixes methodology (how coefficients are calculated) with results (the final numbers) and discussion (why a value is high or low). Present the calculation of FAHP weights, functional coefficients, cost coefficients, and finally the value coefficients for the YS case study. This is the "how." Clearly present the key result—Table 14 showing the value coefficients and identifying components with V<1. The discussion should analyze why certain components (like main construction Y21) have a low V. Discuss the practical optimization measures taken (e.g., "product selection optimization, installation process improvement" mentioned on page 42) in detail here, linking them back to the model's findings. This is the "so what."

5. The conclusion is very brief and simply restates the results. It should be strengthened to: (1) Summarize the key findings; (2) Explicitly state the research contribution (e.g., "This study demonstrates a structured framework for prioritizing cost optimization in complex projects."); (3) Acknowledge the study's limitations more thoroughly (e.g., the model relies on expert judgment for FAHP, the case study is a single project, the "intelligent system" aspect is conceptual); (4) Provide concrete directions for future research (e.g., integrating real-time BIM data, applying machine learning to dynamically update value coefficients, testing the framework on multiple project types).

6. The FAHP calculation process in Sections 2.2 and 3.3.3 is confusing. For example, in the calculation of the criterion layer weights (page 31), the formulas are shown but the intermediate steps for normalizing the judgment matrix are missing, making it difficult to verify the results.

7. Figures 1, 6, and 7 are rudimentary and lack professional quality. Figure 1's data source ("AiMedia Consulting") needs a proper citation. Figures 6 and 7 need to be properly labeled and integrated into the text.

8. The manuscript requires thorough proofreading by a native English speaker or professional editing service to correct grammatical errors and improve sentence fluency (e.g., "commercial complicated project" should be "commercial complex project").

**********

6. PLOS authors have the option to publish the peer review history of their article (what does this mean?). If published, this will include your full peer review and any attached files.

If you choose “no”, your identity will remain anonymous but your review may still be made public.

**Do you want your identity to be public for this peer review?**   For information about this choice, including consent withdrawal, please see our Privacy Policy.

Reviewer #1: No

Reviewer #2: No

**********

[NOTE: If reviewer comments were submitted as an attachment file, they will be attached to this email and accessible via the submission site. Please log into your account, locate the manuscript record, and check for the action link "View Attachments". If this link does not appear, there are no attachment files.]

While revising your submission, please upload your figure files to the Preflight Analysis and Conversion Engine (PACE) digital diagnostic tool, https://pacev2.apexcovantage.com/. PACE helps ensure that figures meet PLOS requirements. To use PACE, you must first register as a user. Registration is free. Then, login and navigate to the UPLOAD tab, where you will find detailed instructions on how to use the tool. If you encounter any issues or have any questions when using PACE, please email PLOS at figures@plos.org. Please note that Supporting Information files do not need this step.

# Author comment: Author response to Decision Letter 1

**DOI:** 10.1371/journal.pone.0343158.r002

21 Oct 2025

Once again, we thank the editor and reviewers for their time and insights. We believe these revisions have significantly strengthened the manuscript’s scientific rigor, clarity, and contribution to the field. We hope the revised version meets the journal’s publication standards.

Attachment Submitted filename: Response to Reviewers.docx

# Peer review: Decision Letter 1

**DOI:** 10.1371/journal.pone.0343158.r003

**Authors:** Tien Anh Tran

26 Nov 2025

PONE-D-25-38715R1

Intelligent system in the cost control of commercial complex projects: data-driven optimization method

PLOS ONE

Dear Dr. Li,

Thank you for submitting your manuscript to PLOS ONE. After careful consideration, we feel that it has merit but does not fully meet PLOS ONE’s publication criteria as it currently stands. Therefore, we invite you to submit a revised version of the manuscript that addresses the points raised during the review process.

==============================

**Kindly address carefully the further comments which the reviewers had indicated the drawbacks. Additionally, the figures of structures should be redrawn clearly to make separated each functional block. There are a lot of typing mistakes in this paper.**

==============================

Please submit your revised manuscript by Jan 10 2026 11:59PM. If you will need more time than this to complete your revisions, please reply to this message or contact the journal office at plosone@plos.org. When you're ready to submit your revision, log on to https://www.editorialmanager.com/pone/ and select the 'Submissions Needing Revision' folder to locate your manuscript file.

Please include the following items when submitting your revised manuscript:

- A rebuttal letter that responds to each point raised by the academic editor and reviewer(s). You should upload this letter as a separate file labeled 'Response to Reviewers'.
- A marked-up copy of your manuscript that highlights changes made to the original version. You should upload this as a separate file labeled 'Revised Manuscript with Track Changes'.
- An unmarked version of your revised paper without tracked changes. You should upload this as a separate file labeled 'Manuscript'.

If you would like to make changes to your financial disclosure, please include your updated statement in your cover letter. Guidelines for resubmitting your figure files are available below the reviewer comments at the end of this letter.

If applicable, we recommend that you deposit your laboratory protocols in protocols.io to enhance the reproducibility of your results. Protocols.io assigns your protocol its own identifier (DOI) so that it can be cited independently in the future. For instructions see: https://journals.plos.org/plosone/s/submission-guidelines#loc-laboratory-protocols. Additionally, PLOS ONE offers an option for publishing peer-reviewed Lab Protocol articles, which describe protocols hosted on protocols.io. Read more information on sharing protocols at https://plos.org/protocols?utm_medium=editorial-email&utm_source=authorletters&utm_campaign=protocols.

We look forward to receiving your revised manuscript.

Kind regards,

Tien Anh Tran

Academic Editor

PLOS ONE

Journal Requirements:

If the reviewer comments include a recommendation to cite specific previously published works, please review and evaluate these publications to determine whether they are relevant and should be cited. There is no requirement to cite these works unless the editor has indicated otherwise.

[Note: HTML markup is below. Please do not edit.]

Reviewers' comments:

Reviewer's Responses to Questions

**Comments to the Author**

1. If the authors have adequately addressed your comments raised in a previous round of review and you feel that this manuscript is now acceptable for publication, you may indicate that here to bypass the “Comments to the Author” section, enter your conflict of interest statement in the “Confidential to Editor” section, and submit your "Accept" recommendation.

Reviewer #2: All comments have been addressed

Reviewer #3: (No Response)

**********

2. Is the manuscript technically sound, and do the data support the conclusions?

The manuscript must describe a technically sound piece of scientific research with data that supports the conclusions. Experiments must have been conducted rigorously, with appropriate controls, replication, and sample sizes. The conclusions must be drawn appropriately based on the data presented.

Reviewer #2: Yes

Reviewer #3: No

**********

3. Has the statistical analysis been performed appropriately and rigorously?

Reviewer #2: Yes

Reviewer #3: No

**********

4. Have the authors made all data underlying the findings in their manuscript fully available?

The PLOS Data policy requires authors to make all data underlying the findings described in their manuscript fully available without restriction, with rare exception (please refer to the Data Availability Statement in the manuscript PDF file). The data should be provided as part of the manuscript or its supporting information, or deposited to a public repository. For example, in addition to summary statistics, the data points behind means, medians and variance measures should be available. If there are restrictions on publicly sharing data—e.g. participant privacy or use of data from a third party—those must be specified.

Reviewer #2: Yes

Reviewer #3: No

**********

5. Is the manuscript presented in an intelligible fashion and written in standard English?

PLOS ONE does not copyedit accepted manuscripts, so the language in submitted articles must be clear, correct, and unambiguous. Any typographical or grammatical errors should be corrected at revision, so please note any specific errors here.

Reviewer #2: Yes

Reviewer #3: No

**********

6. Review Comments to the Author

Please use the space provided to explain your answers to the questions above. You may also include additional comments for the author, including concerns about dual publication, research ethics, or publication ethics. (Please upload your review as an attachment if it exceeds 20,000 characters)

Reviewer #2: The authors' responses are highly appropriate, comprehensive, and effective. They directly address every point of criticism with specific, actionable revisions. The responses demonstrate a clear understanding of my concerns and a strong commitment to improving the manuscript. The proposed changes significantly strengthen the paper by clarifying its methodology, contributions, and technical details.

Reviewer #3: The article addresses important topic for construction industry practice.

However, there are fundamental methodolological mistakes in the paper.

The mistakes mainly result from the misuse of AHP methodology.

For example, Fuzzy AHP is declared. Hwever, crisp AHP assessments are finally applied to get the priorities.

On the other hand, consistency check is inadequate to prioirity estimation technique used (Geometriic Mean technique). This is why Geometric Consistency indcators should be rather applied fror the consistency check and not consistency ratio indicator (CR).

BTW is fuzzy AHP really more confident thatn crisp AHP - see: opinion of AHP inventor (TL.Saaty) about fuzzyfing AHP calculations.

The language and communication merits of the paper are rather poor - there is a need for urgent improvement with this regard.

**********

7. PLOS authors have the option to publish the peer review history of their article (what does this mean?). If published, this will include your full peer review and any attached files.

If you choose “no”, your identity will remain anonymous but your review may still be made public.

**Do you want your identity to be public for this peer review?**   For information about this choice, including consent withdrawal, please see our Privacy Policy.

Reviewer #2: No

Reviewer #3: No

**********

[NOTE: If reviewer comments were submitted as an attachment file, they will be attached to this email and accessible via the submission site. Please log into your account, locate the manuscript record, and check for the action link "View Attachments". If this link does not appear, there are no attachment files.]

To ensure your figures meet our technical requirements, please review our figure guidelines: https://journals.plos.org/plosone/s/figures

You may also use PLOS’s free figure tool, NAAS, to help you prepare publication quality figures: https://journals.plos.org/plosone/s/figures#loc-tools-for-figure-preparation.

NAAS will assess whether your figures meet our technical requirements by comparing each figure against our figure specifications.

# Author comment: Author response to Decision Letter 2

**DOI:** 10.1371/journal.pone.0343158.r004

29 Dec 2025

Thank you for your valuable insights and critical observations on our paper. We highly appreciate your meticulous review of the methodological details and language expression, which provides important directions for improving the quality of our research.

Regarding the misuse of the Fuzzy AHP (FAHP) methodology you pointed out: We acknowledge the inconsistency in our application—while we declared the adoption of FAHP, the final priority calculation relied on crisp AHP assessments. This was an oversight in the research implementation process. In subsequent revisions, we will strictly follow the standard FAHP procedure, integrating fuzzy logic into pairwise comparisons to quantify subjective judgments more scientifically and eliminate the ambiguity in expert evaluations.

On the issue of consistency check: We agree with your view that the consistency ratio (CR) indicator is not suitable for the Geometric Mean technique. We will correct this by applying Geometric Consistency indicators for consistency verification to ensure the reliability of the priority estimation results, in line with the technical requirements of the selected method.

Concerning the confidence comparison between fuzzy AHP and crisp AHP: We have carefully reviewed the opinions of AHP inventor T.L. Saaty on fuzzyfying AHP calculations. In future revisions, we will supplement a detailed discussion on this topic, analyzing the applicability and limitations of FAHP in the context of commercial complex cost control, and explaining why we chose FAHP while responding to relevant academic debates.

Regarding the linguistic presentation of the manuscript: We fully recognize the imperative need for substantial improvements. To this end, we have devoted considerable time to refining the text, optimizing the logical coherence of the expressions, standardizing the use of professional terminology, enhancing the readability and methodological rigor of the paper, and ensuring the effective dissemination of the research findings.

Attachment Submitted filename: Response to Reviewers-2025.12.29.docx

# Peer review: Decision Letter 2

**DOI:** 10.1371/journal.pone.0343158.r005

**Authors:** Tien Anh Tran

19 Jan 2026

PONE-D-25-38715R2

Intelligent system in the cost control of commercial complex projects: data-driven optimization method

PLOS One

Dear Dr. Li,

Thank you for submitting your manuscript to PLOS ONE. After careful consideration, we feel that it has merit but does not fully meet PLOS ONE’s publication criteria as it currently stands. Therefore, we invite you to submit a revised version of the manuscript that addresses the points raised during the review process.

Please submit your revised manuscript by Mar 05 2026 11:59PM. If you will need more time than this to complete your revisions, please reply to this message or contact the journal office at plosone@plos.org. When you're ready to submit your revision, log on to https://www.editorialmanager.com/pone/ and select the 'Submissions Needing Revision' folder to locate your manuscript file.

Please include the following items when submitting your revised manuscript:

- A letter that responds to each point raised by the academic editor and reviewer(s). You should upload this letter as a separate file labeled 'Response to Reviewers'.
- A marked-up copy of your manuscript that highlights changes made to the original version. You should upload this as a separate file labeled 'Revised Manuscript with Track Changes'.
- An unmarked version of your revised paper without tracked changes. You should upload this as a separate file labeled 'Manuscript'.

-->If you would like to make changes to your financial disclosure, please include your updated statement in your cover letter. Guidelines for resubmitting your figure files are available below the reviewer comments at the end of this letter.

If applicable, we recommend that you deposit your laboratory protocols in protocols.io to enhance the reproducibility of your results. Protocols.io assigns your protocol its own identifier (DOI) so that it can be cited independently in the future. For instructions see: https://journals.plos.org/plosone/s/submission-guidelines#loc-laboratory-protocols. Additionally, PLOS ONE offers an option for publishing peer-reviewed Lab Protocol articles, which describe protocols hosted on protocols.io. Read more information on sharing protocols at https://plos.org/protocols?utm_medium=editorial-email&utm_source=authorletters&utm_campaign=protocols.

We look forward to receiving your revised manuscript.

Kind regards,

Tien Anh Tran

Academic Editor

PLOS One

Journal Requirements:

If the reviewer comments include a recommendation to cite specific previously published works, please review and evaluate these publications to determine whether they are relevant and should be cited. There is no requirement to cite these works unless the editor has indicated otherwise.

Please review your reference list to ensure that it is complete and correct. If you have cited papers that have been retracted, please include the rationale for doing so in the manuscript text, or remove these references and replace them with relevant current references. Any changes to the reference list should be mentioned in the rebuttal letter that accompanies your revised manuscript. If you need to cite a retracted article, indicate the article’s retracted status in the References list and also include a citation and full reference for the retraction notice.

**Additional Editor Comments:**

There are some typing mistakes which have been found in this paper. Kindly proofreading this manuscript carefully and correct this mistake.

For example: Figure 2, Kindly adjust this phrase word: Vertical channel.

Figure 4, Missing some words at mid block: Preparat, etc.

so on.

[Note: HTML markup is below. Please do not edit.]

Reviewers' comments:

Reviewer's Responses to Questions

**Comments to the Author**

1. If the authors have adequately addressed your comments raised in a previous round of review and you feel that this manuscript is now acceptable for publication, you may indicate that here to bypass the “Comments to the Author” section, enter your conflict of interest statement in the “Confidential to Editor” section, and submit your "Accept" recommendation.

Reviewer #2: All comments have been addressed

**********

2. Is the manuscript technically sound, and do the data support the conclusions?

The manuscript must describe a technically sound piece of scientific research with data that supports the conclusions. Experiments must have been conducted rigorously, with appropriate controls, replication, and sample sizes. The conclusions must be drawn appropriately based on the data presented.

Reviewer #2: Yes

**********

3. Has the statistical analysis been performed appropriately and rigorously?

Reviewer #2: Yes

**********

4. Have the authors made all data underlying the findings in their manuscript fully available?

The PLOS Data policy requires authors to make all data underlying the findings described in their manuscript fully available without restriction, with rare exception (please refer to the Data Availability Statement in the manuscript PDF file). The data should be provided as part of the manuscript or its supporting information, or deposited to a public repository. For example, in addition to summary statistics, the data points behind means, medians and variance measures should be available. If there are restrictions on publicly sharing data—e.g. participant privacy or use of data from a third party—those must be specified.

Reviewer #2: Yes

**********

5. Is the manuscript presented in an intelligible fashion and written in standard English?

PLOS ONE does not copyedit accepted manuscripts, so the language in submitted articles must be clear, correct, and unambiguous. Any typographical or grammatical errors should be corrected at revision, so please note any specific errors here.

Reviewer #2: Yes

**********

6. Review Comments to the Author

Please use the space provided to explain your answers to the questions above. You may also include additional comments for the author, including concerns about dual publication, research ethics, or publication ethics. (Please upload your review as an attachment if it exceeds 20,000 characters)

Reviewer #2: All comments have been addressed as per my earlier feedback. I don't have additional comments to the authors of the paper.

**********

7. PLOS authors have the option to publish the peer review history of their article (what does this mean?). If published, this will include your full peer review and any attached files.

If you choose “no”, your identity will remain anonymous but your review may still be made public.

**Do you want your identity to be public for this peer review?**   For information about this choice, including consent withdrawal, please see our Privacy Policy.

Reviewer #2: No

**********

[NOTE: If reviewer comments were submitted as an attachment file, they will be attached to this email and accessible via the submission site. Please log into your account, locate the manuscript record, and check for the action link "View Attachments". If this link does not appear, there are no attachment files.]

To ensure your figures meet our technical requirements, please review our figure guidelines: https://journals.plos.org/plosone/s/figures

You may also use PLOS’s free figure tool, NAAS, to help you prepare publication quality figures: https://journals.plos.org/plosone/s/figures#loc-tools-for-figure-preparation.

NAAS will assess whether your figures meet our technical requirements by comparing each figure against our figure specifications.

# Author comment: Author response to Decision Letter 3

**DOI:** 10.1371/journal.pone.0343158.r006

21 Jan 2026

All revisions have been made carefully without altering the original research content and conclusions. We hope the revised manuscript meets the publication requirements. Thank you again for your valuable suggestions, which have helped improve the quality of our paper significantly.

Attachment Submitted filename: Response to Reviewers-2026.01.21.docx

# Editor report: Decision Letter 3

**DOI:** 10.1371/journal.pone.0343158.r007

**Authors:** Tien Anh Tran

2 Feb 2026

Intelligent system in the cost control of commercial complex projects: data-driven optimization method

PONE-D-25-38715R3

Dear Dr. Li,

We’re pleased to inform you that your manuscript has been judged scientifically suitable for publication and will be formally accepted for publication once it meets all outstanding technical requirements.

Within one week, you’ll receive an e-mail detailing the required amendments. When these have been addressed, you’ll receive a formal acceptance letter and your manuscript will be scheduled for publication.

An invoice will be generated when your article is formally accepted. Please note, if your institution has a publishing partnership with PLOS and your article meets the relevant criteria, all or part of your publication costs will be covered. Please make sure your user information is up-to-date by logging into Editorial Manager at Editorial Manager® and clicking the ‘Update My Information' link at the top of the page. For questions related to billing, please contact billing support.

If your institution or institutions have a press office, please notify them about your upcoming paper to help maximize its impact. If they’ll be preparing press materials, please inform our press team as soon as possible -- no later than 48 hours after receiving the formal acceptance. Your manuscript will remain under strict press embargo until 2 pm Eastern Time on the date of publication. For more information, please contact onepress@plos.org.

Kind regards,

Tien Anh Tran

Academic Editor

PLOS One

Additional Editor Comments (optional):

Reviewers' comments:

# Editor report: Acceptance letter

**DOI:** 10.1371/journal.pone.0343158.r008

**Authors:** Tien Anh Tran

PONE-D-25-38715R3

PLOS One

Dear Dr. Li,

I'm pleased to inform you that your manuscript has been deemed suitable for publication in PLOS One. Congratulations! Your manuscript is now being handed over to our production team.

At this stage, our production department will prepare your paper for publication. This includes ensuring the following:

* All references, tables, and figures are properly cited

* All relevant supporting information is included in the manuscript submission,

* There are no issues that prevent the paper from being properly typeset

You will receive further instructions from the production team, including instructions on how to review your proof when it is ready. Please keep in mind that we are working through a large volume of accepted articles, so please give us a few days to review your paper and let you know the next and final steps.

Lastly, if your institution or institutions have a press office, please let them know about your upcoming paper now to help maximize its impact. If they'll be preparing press materials, please inform our press team within the next 48 hours. Your manuscript will remain under strict press embargo until 2 pm Eastern Time on the date of publication. For more information, please contact onepress@plos.org.

You will receive an invoice from PLOS for your publication fee after your manuscript has reached the completed accept phase. If you receive an email requesting payment before acceptance or for any other service, this may be a phishing scheme. Learn how to identify phishing emails and protect your accounts at https://explore.plos.org/phishing.

If we can help with anything else, please email us at customercare@plos.org.

Thank you for submitting your work to PLOS ONE and supporting open access.

Kind regards,

PLOS ONE Editorial Office Staff

on behalf of

Professor Tien Anh Tran

Academic Editor

PLOS One
